# Thin Film Composite Membranes with Regulated Crossover and Water Migration for Long‐Life Aqueous Redox Flow Batteries

**DOI:** 10.1002/advs.202206888

**Published:** 2023-05-13

**Authors:** Rui Tan, Anqi Wang, Chunchun Ye, Jiaxi Li, Dezhi Liu, Barbara Primera Darwich, Luke Petit, Zhiyu Fan, Toby Wong, Alberto Alvarez‐Fernandez, Mate Furedi, Stefan Guldin, Charlotte E. Breakwell, Peter A. A. Klusener, Anthony R. Kucernak, Kim E. Jelfs, Neil B. McKeown, Qilei Song

**Affiliations:** ^1^ Department of Chemical Engineering Imperial College London London SW7 2AZ UK; ^2^ EaStChem School of Chemistry University of Edinburgh Edinburgh EH9 3FJ UK; ^3^ Department of Chemical Engineering University College London London WC1E 7JE UK; ^4^ Department of Chemistry Molecular Sciences Research Hub Imperial College London London W12 0BZ UK; ^5^ Shell Global Solutions International B.V. Energy Transition Campus Amsterdam HW Amsterdam Grasweg 31 1031 The Netherlands

**Keywords:** energy storage, ion‐selective membranes, microporous polymers, redox flow batteries

## Abstract

Redox flow batteries (RFBs) are promising for large‐scale long‐duration energy storage owing to their inherent safety, decoupled power and energy, high efficiency, and longevity. Membranes constitute an important component that affects mass transport processes in RFBs, including ion transport, redox‐species crossover, and the net volumetric transfer of supporting electrolytes. Hydrophilic microporous polymers, such as polymers of intrinsic microporosity (PIM), are demonstrated as next‐generation ion‐selective membranes in RFBs. However, the crossover of redox species and water migration through membranes are remaining challenges for battery longevity. Here, a facile strategy is reported for regulating mass transport and enhancing battery cycling stability by employing thin film composite (TFC) membranes prepared from a PIM polymer with optimized selective‐layer thickness. Integration of these PIM‐based TFC membranes with a variety of redox chemistries allows for the screening of suitable RFB systems that display high compatibility between membrane and redox couples, affording long‐life operation with minimal capacity fade. Thickness optimization of TFC membranes further improves cycling performance and significantly restricts water transfer in selected RFB systems.

## Graphical abstract

Polymers of intrinsic microporosity (PIMs) are promising as next‐generation ion‐selective membranes in redox flow batteries for large‐scale long‐duration energy storage. By optimizing the thickness of the selective PIM layer of thin film composite membranes, electrolyte crossover and water migration could be effectively mitigated, leading to better cycling stability of batteries.

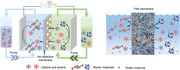

## 1 Introduction

The development of renewable energy and efficient energy storage technologies is crucial in achieving net‐zero emissions and global carbon neutrality.^[^ ^1^ ^]^ Redox flow batteries (RFBs) are a promising technology for large‐scale stationary energy storage due to their appealing features including cost‐effectiveness, safety, and high efficiency.^[^ ^2^ ^]^ RFBs comprise two half‐cell electrolytes separated by a conductive and selective membrane that facilitates the transport of charge‐balancing ions and minimizes the cross‐mixing of redox species. RFB systems possess decoupled nature of power and energy and cost‐effectiveness, which are inherently well suited for mega‐to‐gigawatt hour‐scale energy storage compared with other batteries, for example, conventional lithium‐ion batteries.^[^ ^3^ ^]^ The capacity retention and power of RFBs are substantially affected by the performance of membranes.^[^ ^4^ ^]^ Therefore, it is desirable to design and develop low‐cost, highly conductive, and selective membranes, particularly for RFB chemistries that employ metal ion‐based^[^ ^5^ ^]^ and organic molecule‐based^[^ ^6^ ^]^ redox‐active species.

Perfluorinated sulfonic acid (PFSA) membranes, for example, Nafion, are polymer electrolyte membranes with superior chemical stability and ionic conductivity that have been widely used in electrochemical devices including RFBs.^[^ ^7^ ^]^ However, their high cost and poor selectivity for small charge‐balancing ions over larger redox‐active species are limiting the widespread deployment and commercialization of RFBs. Alternative membrane materials have been developed with a focus on low‐cost hydrocarbon‐based ion exchange membranes.^[^ ^8^ ^]^ These fluorocarbon and hydrocarbon ion exchange membranes, however, are limited by the trade‐off between ionic conductivity and selectivity, and suffer from fast contra‐permeation of redox species and the net volumetric transfer of water in aqueous redox flow batteries.^[^ ^9^ ^]^

Water migration is an important yet often overlooked challenge for the long‐term operation of RFB systems. The net volumetric change of the electrolytes can result in the precipitation of redox species on one side and flooding of the electrolyte reservoir on the other, causing operational difficulties and requiring sophisticated water management in RFB systems.^[^ ^10^ ^]^ The preferential water transfer across membranes is driven by multiple factors including osmotic pressure, hydraulic pressure, and the migration of charged species. From the engineering perspective, the net volumetric change can be regulated by adjusting liquid flow rates, tuning the concentrations of redox‐active species, or adding the balancing additives. However, the intrinsic membrane properties also determine mass transport and water migration. Particularly, the transfer of water carried by charged redox‐active species is a major factor contributing to the undesired water migration, while the permeation rate of redox‐active species is a function of solute permeability and membrane thickness following Darcy's law.^[^ ^11^ ^]^ Therefore, there are two approaches that may provide effective solutions to water migration, one by designing new membrane materials that show low values of intrinsic permeability for redox active species, and the other by optimizing the membrane thickness. Lu group demonstrated the development of charge‐reinforced ion‐selective membranes with effective mitigation of crossover of polysulfides by negatively charged carbon layer, as well as free water migration due to the hydrophobicity and narrow size of water channels in membranes.^[^ ^12^ ^]^

**Figure 1:**
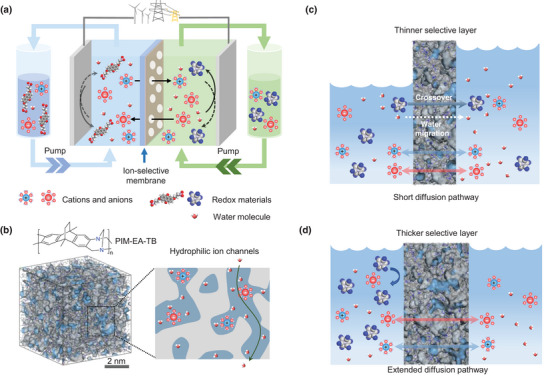
Ion‐selective PIM membranes for RFBs. a) Schematic illustration of an operating aqueous RFB. b) 3D view of an amorphous PIM‐EA‐TB cell generated by protocol and reported in our previous work,^[^ ^17^ ^]^ and schematic showing the water‐assisted hydrophilic ion channels. c) Severe net volumetric transfer in an RFB with a thinner selective membrane. d) Managed net volumetric transfer in an RFB with a thicker selective membrane.

Polymers of intrinsic microporosity (PIMs) have emerged as a new class of membrane materials for ionic and molecular transport that can break the trade‐off between permeability and selectivity due to their rigidity and intrinsic sub‐nanometer pores.^[^ ^13^ ^]^ PIM membranes have demonstrated promising performance in a range of electrochemical devices, such as solid‐state batteries,^[^ ^14^ ^]^ lithium–sulfur batteries,^[^ ^15^ ^]^ and redox flow batteries^[^ ^16^ ^]^ (**Figure** **1**a). Of particular interest are Tröger's base PIMs (TB‐PIMs) that can be solution‐cast onto low‐cost porous supports affording thin film composite (TFC) membranes with tuneable thickness.^[^ ^17^ ^]^ In comparison to the dense membranes, using TFC membranes in RFB could effectively decrease the areal‐specific resistance and boost the device performance in terms of energy efficiency and power. Furthermore, TFC membranes are cost‐effective and can be scaled up using industrial membrane manufacturing processes. TB‐PIM TFC membranes have been utilized as ion conductive and selective membranes in alkaline aqueous organic RFBs, but these membranes with sub‐micrometer‐thick PIM layer suffer from severe water migration and fast redox‐species crossover, necessitating further membrane development (Figure 1b,c).

In this work, we propose a simple and novel strategy to tune the transport performance of PIM‐EA‐TB TFC membranes by thickness optimization, and employ these membranes to mitigate the net volumetric transfer of electrolytes in selected RFB chemistries operated at near neutral‐pH (Figure 1d). Transport of water, ions, and redox‐active molecules are systematically investigated for these TFC membranes with PIM layer thickness ranging from 0.3 to 12 µm, establishing the correlation between the transport properties and selective layer thickness. The permeation of redox species is reduced by 1–2 orders of magnitude and the water transfer can be effectively controlled by thickness optimization. These PIM‐EA‐TB TFC membranes show high ionic conductivity and low permeation rates of redox‐active species, and enable long‐life cycling operation of RFBs when paired with 2,6‐DPPAQ||K_4_Fe(CN)_6_ redox couple, showing low capacity decay (≈0.005% per cycle) over 4500 cycles. Our strategy may serve as an alternative approach to addressing the electrolyte and water migration issues in RFBs to further enhance their efficiency and longevity.

## 2 Results and Discussion

### 2.1 Membrane Preparation and Characterization

**Figure 2:**
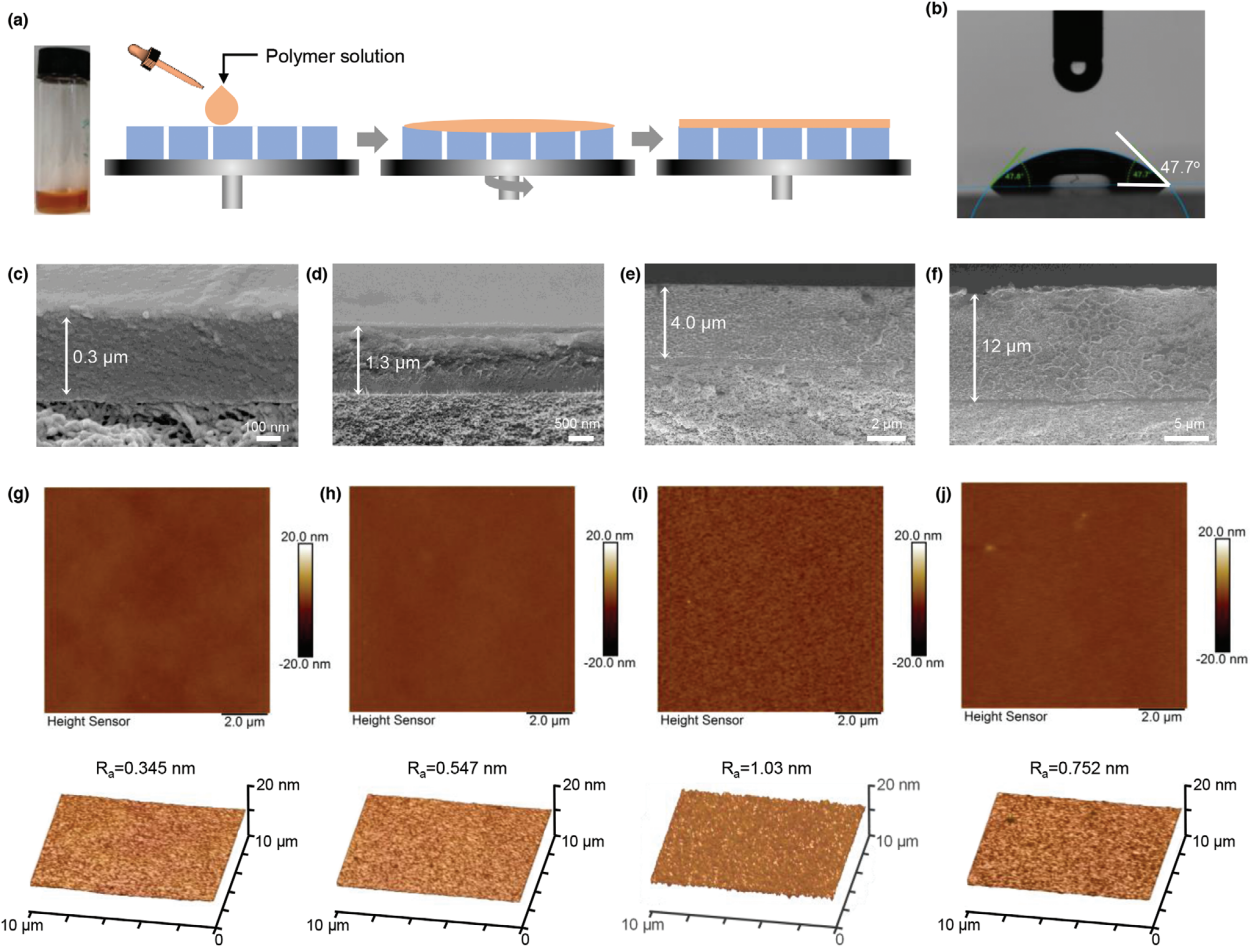
Membrane preparation and characterization. a) Schemes showing the preparation of TFC membranes with a PIM polymer solution by a spin‐coating method onto a porous substrate. b) Water contact angle of PIM‐EA‐TB‐0.3. c–f) Cross‐sectional morphologies of PIM‐EA‐TB‐0.3, PIM‐EA‐TB‐1.3, PIM‐EA‐TB‐4.0 and PIM‐EA‐TB‐12. g–j) AFM images showing the surface morphologies (top) and roughness (bottom) of PIM‐EA‐TB‐0.3, PIM‐EA‐TB‐1.3, PIM‐EA‐TB‐4.0, and PIM‐EA‐TB‐12.

PIM‐EA‐TB was selected as a prototypical polymer to demonstrate our proposed approach of thickness engineering. PIM‐EA‐TB possesses highly interconnected micropores with an apparent BET surface area of 1028 m^2^ g^−1^ owing to the hindered packing of the highly rigid, contorted polymer chains,^[^ ^13c^ ^]^ while the “built‐in” hydrophilic TB groups ensure the percolation of water channels in aqueous electrolytes to afford well‐defined ion transport pathways. PIM‐EA‐TB was dissolved in chloroform at varied concentrations (2.0, 4.0, 6.0, and 8.0 wt.%) and these solutions were spin‐coated onto porous polyacrylonitrile (PAN) supports to produce TFC membranes with varied thickness (**Figure** **2**a). The PAN substrate had abundant meso‐/macro pores (Figure S1, Supporting Information) so that it was highly permeable to guest solutes and functioned as a robust support for hydrophilic PIM‐EA‐TB thin films (Figure 2b). The thicknesses and morphologies of PIM‐EA‐TB TFC membranes were investigated by a field emission scanning electron microscope (SEM) and an atomic force microscope (AFM). As shown in Figure 2c–f, these membranes featured selective layers of varied thicknesses (0.3–12 µm). For simplicity, these composite membranes are denoted as PIM‐EA‐TB‐0.3, PIM‐EA‐TB‐1.3, PIM‐EA‐TB‐4.0 and PIM‐EA‐TB‐12 based on the thicknesses of selective layers (e.g., PIM‐EA‐TB‐4.0 for a TFC membrane with a 4‐µm selective layer). The nonlinear increase of selective layer thickness might be attributed to the solution viscosity change along with the increased concentration of PIM solutions. We demonstrated our concept and proved the feasibility of our strategy by focusing on these four typical target samples. Both cross‐sectional and surface SEM images showed dense and defect‐free morphologies, which were also confirmed by AFM images (Figure 2g–j). As‐prepared membranes displayed smooth surfaces with surface roughness in the range of 0.468–1.47 nm (R_q_) and 0.345–1.03 nm (R_a_) (Table S1, Supporting Information). These results confirmed the successful fabrication of TFC membranes with defect‐free PIM‐EA‐TB selective layers.

**Figure 3:**
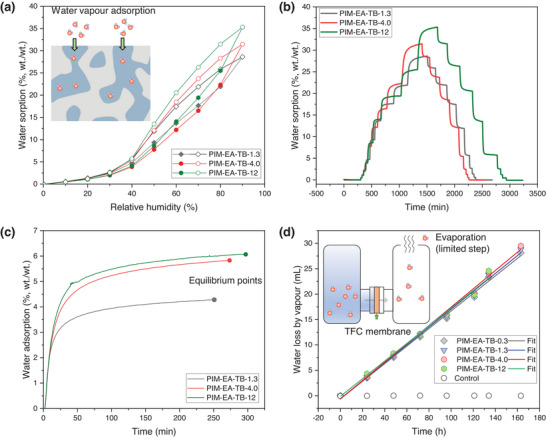
Dynamic water vapor sorption. a) Dynamic vapor sorption isotherms at 0–100% relative humidity (RH). b) Dynamic vapor sorption profiles against testing time. c) Vapor sorption profiles of PIM‐EA‐TB‐1.3, PIM‐EA‐TB‐4.0, and PIM‐EA‐TB‐12 from 70% RH to 80% RH. d) Pervaporation measurements at 70 °C over 165 h. Selective layers within TFC membranes faced the liquid water side while the supports were exposed to the open air at 20% RH.

Hydrophilicity and water permeation for PIM‐EA‐TB TFC membranes were evaluated by using contact angle measurements, sorption, pervaporation, and nanofiltration tests. PIM‐EA‐TB with hydrophilic Tröger's base units show high wettability, as evidenced by the low contact angles in water and alkaline solutions, for example, PIM‐EA‐TB‐4.0 in water 44.1^o^ and in 1 m NaOH solution 55.0^o^ (Figure S2a, Supporting Information). The water contact angles gradually reduced over time (Figure S2b, Supporting Information), indicating the certain compatibility between water and the membranes, which is a critical factor for the formation of continuous water channels.^[^ ^18^ ^]^ Subsequently, we proceeded to evaluate the kinetics and permeances of water vapor and liquid water transport in and across membranes as well as the geometry change of wet membranes via a variety of techniques. As shown in the dynamic vapor sorption (**Figure** **3**a,b; Figure S3, Supporting Information), thicker selective PIM‐EA‐TB layers can adsorb more water, for example, 31.4% (wt./wt.) for PIM‐EA‐TB‐4.0 and 35.3% (wt./wt.) for PIM‐EA‐TB‐12, suggesting the re‐arrangement of polymer chains in wet thicker membranes that possibly generate additional nanoscale pores for the adsorption of water molecules via the capillary effect. The kinetics of water in membranes were qualitatively compared during 70%–80% RH (Figure 3c), in which thicker PIM‐EA‐TB required a longer time to reach equilibrium, indicating a sluggish water saturation process. All PIM‐EA‐TB membranes exhibited identical pervaporation rates of ≈ 0.17–0.18 mL h^−1^, attributing to the slow evaporation of water molecules which is the rate‐limiting step (Figure 3d). The water vapor sorption and induced geometry change of PIM‐EA‐TB thin films with varied thicknesses were also investigated using ellipsometry tests (Figure S4, Supporting Information).

**Figure 4:**
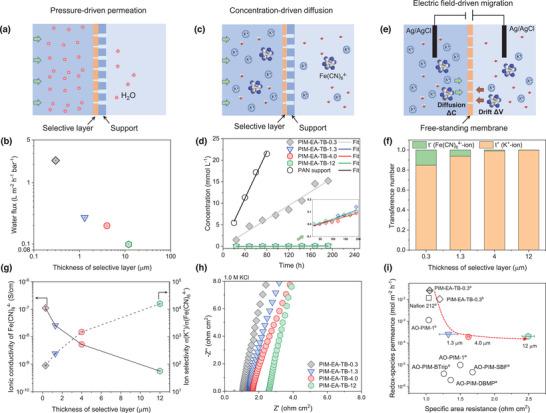
Water and ion transport through PIM membranes. a) Water permeation in a pressure‐driven process. b) Water permeance for the membranes with the varied thicknesses of selective layers. c) Illustration of the concentration‐driven dialysis. d) Crossover diffusion of K_4_Fe(CN)_6_ through membranes. e) Schematic showing the convection‐diffusion driven by the salinity concentration and electrical field. f) Cation selectivity. g) Ionic conductivity of large anions and the selectivity of cations to anions. h) Ion transport resistance of PIM‐EA‐TB TFC membranes in 1 m KCl solutions at 30 °C. i) Comparison of specific area resistance and redox‐species crossover for different membranes, including PIM‐EA‐TB TFC with varied thicknesses, AO‐PIM‐1^b^, and PIM‐EA‐TB‐0.3^b^,^[^ ^17^ ^]^ Nafion 212 and AO‐PIM series (AO‐PIM‐1^b^, AO‐PIM‐SBF^b^, AO‐PIM‐BTrip^b^, and AO‐PIM‐DBMP^b^).^[^ ^16b^ ^]^ Redox species: K_4_Fe(CN)_6_. ^a^Resistance in 1 m KCl; ^b^Resistance in 1 m KOH.

Liquid water permeation across membranes is a more critical property that directly determines the water migration in RFB scenarios. We, therefore, measured the water permeances through these membranes using a pressure‐driven nanofiltration cell at 30 bar (**Figure** **4**a). The water permeance/flux significantly decreased with increasing the thickness of selective layers from 0.3 to 12 µm (Figure 4b). For example, PIM‐EA‐TB‐4.0 with a 4‐µm selective layer presented a significantly lower water flux of 0.20 L m^−2^ h^−1^ bar^−1^ than that of the TFC membrane with a thickness of 0.3 µm (2.30 L m^−2^ h^−1^ bar^−1^). Hence, the thick PIM‐EA‐TB TFC membranes with sluggish water saturation and reduced permeance may play an important role in mitigating the water migration and limiting the crossover of redox species.

### 2.2 Selective Transport of Ions and Redox Species

The diffusion of redox species through the PIM‐EA‐TB TFC membranes was measured by both concentration‐driven dialysis (Figure 4c) and a convection method with driving forces of salinity concentration and electrical field (Figure 4e). A K_4_Fe(CN)_6_ solution (0.1 m) was used as the feed solution and deionized water was employed as the permeate solution, from which the permeated Fe(CN)_6_^4−^‐ions were quantified using inductively coupled plasma optical emission spectroscopy (ICP‐OES). The permeation rates of Fe(CN)_6_^4−^ through PIM‐EA‐TB‐1.3, PIM‐EA‐TB‐4.0 and PIM‐EA‐TB‐12 are 0.25, 0.19 and 0.20 mmol m^−2^ h^−1^, respectively, much lower than that of thin PIM‐EA‐TB‐0.3 (25.8 mmol m^−2^ h^−1^) (Figure 4d). Cation selectivity of PIM‐EA‐TB TFC membranes was investigated with K_4_Fe(CN)_6_, KOH, and KCl solutions (Figure 4f and Table S2, Supporting Information). Based on the results of the transference number for KCl solutions, we can conclude that the principle for selective ion transport is dominated by the size‐exclusion mechanism rather than the Donnan exclusion effect since the PIM‐EA‐TB (pK_a_≈4) could not be protonated to produce charged functional groups in alkaline and near neutral conditions.^[^ ^19^ ^]^ With the thickness of the selective layers increased from 0.3 to 12 µm, the transference number of K^+^ cations increased from 0.847 to nearly 1 for K_4_Fe(CN)_6_ solutions, suggesting the mobility of large‐sized Fe(CN)_6_^4−^‐ions was restricted by the extended tortuous ion transport pathways. The ion selectivity of K^+^ over Fe(CN)_6_^4−^ for PIM‐EA‐TB‐12 was two orders of magnitude higher than that of PIM‐EA‐TB‐0.3 (Figure 4g). The immobilization of Fe(CN)_6_^4−^‐ions was also evidenced by the mobility ratios of K^+^ over Fe(CN)_6_^4−^ increasing from 1.29 to 1.70 (Figure S5, Supporting Information). These results proved that thicker PIM‐EA‐TB TFC membranes exhibited reduced crossover of redox‐active species and improved cation selectivity, which would be significant for prohibiting redox‐species crossover in redox flow batteries.

We measured the ion transport resistance of our PIM‐EA‐TB TFC membranes in 1 m KCl solutions in the temperature range of 20–70 °C using electrochemical impedance spectroscopy (EIS) (Figure 4h; Figures S6–S8, Supporting Information). Compared with PIM‐EA‐TB‐0.3, thicker PIM‐EA‐TB‐4.0 presented a relatively high overall ohmic resistance of 1.63 Ω cm^2^ with a resistance of 0.75 Ω cm^2^ as calculated by subtracting the resistance of the hydrolyzed PAN support (Figure S7, Supporting Information). Nevertheless, the thicker PIM‐EA‐TB TFC membranes still maintained high ionic conductivity in neutral‐pH salt solutions at room temperature, with values in the range 1.9–3.3 mS cm^−1^ that are comparable to that of Nafion 212 in 1 m KCl (5.4 mS cm^−1^)^[^ ^16b^ ^]^ (Figure S8, Supporting Information). The low activation energy of ion transport in these PIM‐EA‐TB membranes (3.34–12.5 kJ mol^−1^) suggests that charge‐balancing ions can smoothly migrate in the interconnected tortuous micropores.

Area‐specific resistance (ASR) and permeability of redox species are two critical membrane parameters for the efficient operation of redox flow batteries. Figure 4i shows a comparison of the combination of redox‐species permeation rates and area‐specific resistances for these membranes. Though thicker PIM‐EA‐TB TFC membranes exhibit relatively higher ASR (1.48 Ω cm^2^ for PIM‐EA‐TB‐1.3; 1.63 Ω cm^2^ for PIM‐EA‐TB‐4.0), their selective performance against redox species is superior to our previously reported PIM‐EA‐TB TFC membranes (1.20 Ω cm^2^; 10.4 mmol m^−2^ h^−1^)^[^ ^16^, ^17^ ^]^ and the benchmark Nafion 212 membrane (1.07 Ω cm^2^; 11.7 mmol m^−2^ h^−1^).^[^ ^16^, ^17^ ^]^ Such improved performance suggests that thick PIM‐EA‐TB TFC membranes with extended diffusion channels allow the fast transport of ions while slowing down the crossover of water and redox species, which is beneficial for the stable operation of RFBs.

### 2.3 Screening of Redox Chemistries and Demonstration of Long‐Cycling RFBs

**Figure 5:**
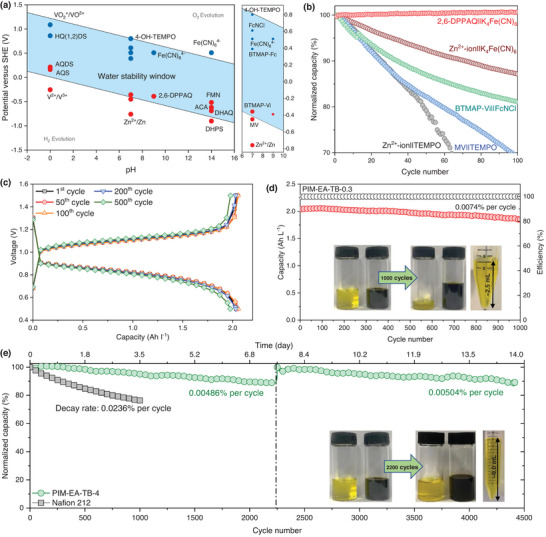
Redox flow battery performance. a) Summary of recently reported redox species with their redox potentials at the pH 0–14. The blue zone represents the electrochemical stability window of water, which was derived from the Nernst equation. b) Comparison of cycling performance of paired redox species with PIM‐EA‐TB‐0.3 in near‐/neutral conditions. c) Typical charging–discharging profiles of a 2,6‐DPPAQ||K_4_Fe(CN)_6_ battery with a PIM‐EA‐TB‐0.3 membrane. d) Long‐term cycling performance of a 2,6‐DPPAQ||K_4_Fe(CN)_6_ battery with a PIM‐EA‐TB‐0.3 membrane. Inset shows the volume of electrolytes before and after 1000 cycles. e) Long‐term cycling performance of a 2,6‐DPPAQ||K_4_Fe(CN)_6_ battery with a PIM‐EA‐TB‐4.0 membrane. Inset shows the volume of electrolytes before and after 2200 cycles.

The compatibility between redox couples and membranes is of crucial importance for realizing the full potential of our membranes and enabling high device performance. Owing to the instability of PAN supports in alkaline solutions, we selected redox species that are generally used in benign near‐neutral pH aqueous solutions. **Figure** **5**a summarizes the redox potentials of recently reported redox species^[^ ^6^, ^20^ ^]^ at the pH 0–16 and the electrochemical stability window of water. Based on this plot, anodic materials, including Zn^2+^‐ion, methyl viologen, BTMAP‐Vi, and 2,6‐DPPAQ, were selected and paired with cathodic materials, TEMPO, ferri/ferrocyanide, and FcNCl according to the redox potentials and molecular sizes of these redox species (Figure S9a, Supporting Information). As a consequence, five pairs of redox chemistries were selected to develop near‐/neutral aqueous RFBs with PIM‐EA‐TB TFC membranes (Figure S9b, Supporting Information). For the sake of experimental simplicity, PIM‐EA‐TB‐0.3 was used for fast screening of the most compatible redox species in operating devices.

The key performance indicators of device performance were evaluated in terms of the working plateau, capacity loss rate, and cycle life (Figure 5b). Though the flow cell with Zn^2+^‐ion||TEMPO delivered a high output voltage of 1.51 V (Figures S10 and S11a,b, Supporting Information), the capacity retention was only ≈78% over 50 cycles at 20 mA cm^−2^ due to the fast permeation of small‐sized TEMPO radicals (6.5×8.7×9.1Å^3^)^[^ ^21^ ^]^ through the PIM‐EA‐TB‐0.3 membrane. Similarly, MV||TEMPO with an output voltage of 1.20 V underwent fast capacity decay and low‐capacity retention of ≈86% over 50 cycles at 20 mA cm^−2^ (Figure S12, Supporting Information). Replacing the TEMPO species with larger ferrocyanide mitigated the crossover issue and enhanced battery cyclability, which was demonstrated in a Zn||K_4_Fe(CN)_6_ battery with ≈77% capacity retention after 800 cycles (Figure 5b; Figure S11c,d, Supporting Information). However, this battery still exhibited noticeable capacity decay, and despite the lack of metal dendrites formed on the surface of anodes (Figure S11e,f, Supporting Information). We assume that the negative potential of Zn^2+^/Zn might induce irreversible water electrolysis and cause capacity loss over long‐term operation, making this species inappropriate for anodic materials. Besides, the reaction between Zn^2+^ and Fe(CN)_6_^4−^ results in the precipitation of zinc hexacyanoferrate (Prussian blue analogues), leading to the loss of redox active species and further deteriorating the cycling performance of this battery. Positively charged BTMAP‐Vi with a large molecular size was used as the substitute for Zn anode, but this redox species undesirably contaminated the PIM‐EA‐TB‐0.3 membrane and deteriorated battery cyclability (Figure S13, Supporting Information), due to the interaction between Tröger's base and positively charged species.^[^ ^22^ ^]^ Negatively charged 2,6‐DPPAQ was therefore selected as the electrolyte due to its relatively higher potential (−0.39 vs SHE within the water stable window) and relatively large molecular size. Flow batteries using a 2,6‐DPPAQ||K_4_Fe(CN)_6_ redox pair and a PIM‐EA‐TB‐0.3 membrane exhibited a remarkably improved cycling stability with a low capacity decay of 0.0074% per cycle over 1000 cycles (≈1.9% per day), which was superior to the performance of benchmark Nafion 212 (0.0236% per cycle) (Figure 5c–e) and much better than our first demonstration of TFC membrane (3.2% per day).^[^ ^17^ ^]^ However, batteries using PIM‐EA‐TB‐0.3 suffered from a significant water migration from catholytes to anolytes with a rate of ≈1.3 mL per day as shown in Figure 5d, which will become a critical issue for practical RFB applications.

To tackle the issue of electrolyte transfer and further improve battery cycling stability, we prepared PIM‐EA‐TB‐4.0 with a thicker selective layer of 4.0 µm, which has demonstrated comparable ion conductivity and significantly limited permeation of water and electrolyte. As a result, PIM‐EA‐TB‐4.0 with an ASR of 2.18 Ω cm^2^ (Figure S14, Supporting Information), enabled a long‐cycling RFB with an extremely low decay rate of 0.00486–0.00504% per cycle over 4500 cycles (≈14 days, Figure 5e). The membranes recovered from the cycled cell were crack‐free and clean without evident absorption and fouling by the electrolytes (Figure S15, Supporting Information). The battery recovered to the original capacity when after cycling the used electrolytes were replaced with fresh electrolytes (Figure 5e), suggesting that the capacity decay was not due to the degradation or fouling of the membrane. Notably, the net volumetric loss of electrolyte for PIM‐EA‐TB‐4.0 was only 0.08 mL per day, about one order of magnitude lower than that of PIM‐EA‐TB‐0.3.

**Figure 6:**
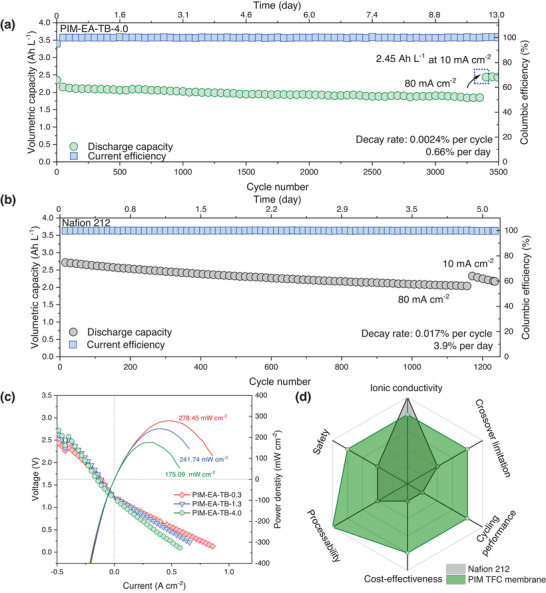
Cycling stability of redox flow battery. a) Long‐term cycling performance of a 2,6‐DPPAQ||K_4_Fe(CN)_6_ battery with (a) a PIM‐EA‐TB‐4.0 membrane and b) a Nafion 212 membrane at 80 and 10 mA cm^−2^. c) Power density of a 2,6‐DPPAQ||K_4_Fe(CN)_6_ battery with a PIM‐EA‐TB TFC membranes and 1 m redox species. d) Comparison of key parameters for PIM TFC and Nafion 212 membranes.

The practical rate of capacity loss for the 2,6‐DPPAQ||K_4_Fe(CN)_6_ battery with a PIM‐EA‐TB‐4.0 membrane was evaluated by the battery rate tests (**Figure** **6**a). The capacity returned to 2.45 Ah L^−1^ at 10 mA cm^−2^ and the capacity retention was 91.4% over 3500 cycles, equal to a decay rate of 0.0024% per cycle. Such decay rate was 10 times lower than that of a benchmark Nafion 212 (0.017% per cycle) tested by the same protocol (Figure 6b). At a current density of 10 mA cm^−2^, the coulombic efficiency, voltage efficiency and energy efficiency were 99.9%, 92.4%, and 92.3%, respectively, after the battery underwent 3390 cycles at 80 mA cm^−2^ and subsequent 100 cycles at 10 mA cm^−2^ (Figure S16, Supporting Information). This remarkable ratability confirmed the stability of our developed membranes in selected aqueous organic RFBs. At 80 mA cm^−2^, the energy efficiency of this cell maintained a value high up to ≈65% with a voltage hysteresis of 0.38 V, slightly lower than that of Nafion 212 (≈72%) over a long cycling duration as shown in Figure S17 (Supporting Information). After cycling, the electrolytes were further tested by cyclic voltammetry to evaluate the crossover of redox species. The anolyte of the battery using a Nafion 212 membrane exhibited a high peak current of 0.35 mA, equal to 1.1×10^−2^ mmol, which could be attributed to the redox reaction of a large amount of permeated K_4_Fe(CN)_6_. In comparison, PIM‐EA‐TB‐4.0 restricted the crossover of K_4_Fe(CN)_6_ to 1.4×10^−3^ mmol as shown by a low peak current of ≈0.04 mA (Figure S18, Supporting Information). The electrolyte migration and electrochemical performance were further investigated in the high‐concentration 2,6‐DPPAQ||K_4_Fe(CN)_6_ batteries using PIM‐EA‐TB‐4.0 TFC membrane in the open air (Figure S19, Supporting Information). Over the charging duration of 1 h, the established battery delivered a high capacity of 15.1 Ah L^−1^ and a minimal volume change of ≈ 0.1 mL over 100 cycles at 20 mA cm^−2^ for 200 h. Hence, the synergetic integration of PIM‐EA‐TB‐4.0 with 2,6‐DPPAQ||K_4_Fe(CN)_6_ redox species significantly limited the redox‐species crossover and mitigated the net volumetric transfer as well as enhanced the cycling lifetime for near‐neutral RFB systems.

The ion‐transport impedance and power densities of 2,6‐DPPAQ||K_4_Fe(CN)_6_ flow batteries with these PIM‐EA‐TB TFC membranes were systematically studied at different states‐of‐charge (SOC) (Figures S20 and S21, Supporting Information). Charged batteries generally possess a reduced ohmic resistance compared with the resistance at 0% SOC. For example, a battery with a PIM‐EA‐TB‐4.0 membrane has a lower overall ASR of ≈1.94 Ω cm^2^ at 20%, 50%, and 100% SOC, than the resistance of the battery at 0% SOC (2.18 Ω cm^2^), suggesting an activation process for ion conduction in PIM‐EA‐TB TFC membranes. Batteries with 0.1 m K_4_Fe(CN)_6_ redox species delivered power densities of 152.8, 145.4 and 89.3 mW cm^−2^ for PIM‐EA‐TB‐0.3, PIM‐EA‐TB‐1.3, and PIM‐EA‐TB‐4.0 membranes, respectively (Figure S21a, Supporting Information). Increasing the concentration of used redox species boosted the battery power, for example, 175.1 mW cm^−2^ for PIM‐EA‐TB‐4.0 with 1.0 m K_4_Fe(CN)_6_ redox species (Figure 6c,d compares the key parameters for PIM‐EA‐TB TFC and Nafion 212 membranes. PIM‐EA‐TB TFC membranes generally possess comparable electrochemical properties and superior cycling performance to Nafion 212 due to the regulated mass transfer and electrolyte migration (Tables S3 and S4, Supporting Information). Particularly, the estimated cost of PIM‐EA‐TB TFC membranes is only £0.7 m^−2^ (0.3‐µm‐layer) and £9.2 m^−2^ (4.0‐µm‐layer) and solution‐processable PIM‐EA‐TB can be inherently appropriate for the industrial membrane manufacturing using a roll‐to‐roll technology, making it affordable for the application in large‐scale RFB stacks.

Despite the improved cycling life, our developed TFC membranes still present relatively high ion transport resistance, which is mainly attributed to the resistance from PAN support (≈

0.88 Ω cm^2^). These membranes also limit the applications in benign near‐/neutral solutions due to the hydrolysis of unstable PAN supports in alkaline and acidic conditions. Besides the development of alternative support materials with low resistance and sufficient chemical stability, it is necessary to develop alternative PIM chemistries that are hydrophilic, electro‐/chemically stable, and can be easily processed, which is important for further expanding applications of these membranes to other battery chemistries operated in harsher conditions.

## 3 Conclusion

This work demonstrated a facile approach to regulating the electrolyte crossover and stabilizing the RFB cycling performance by tuning the selective‐layer thickness of PIM‐EA‐TB TFC membranes. PIM‐EA‐TB TFC membranes with thicker selective layers possess extended tortuous diffusion pathways that reduce water migration and redox‐species crossover. The use of these membranes was proved by pairing with a variety of redox chemistries in near‐neutral conditions. The 2,6‐DPPAQ||K_4_Fe(CN)_6_ redox species combined with PIM‐EA‐TB TFC membranes synergistically afforded a long cycling life RFB system with restricted crossover and water migration. The development of low‐cost TFC membranes to replace the conventional perfluorinated Nafion membranes is of great significance for reducing the capital costs of flow battery stack and electrochemical energy storage. We expect that this approach to the preparation of thin film composite membranes and the regulation of water migration and crossover could be extended to the fabrication of better membranes, offering a simple, low‐cost, and enabling technology for low‐cost high‐performance RFBs for large‐scale long‐duration energy storage.

## Conflict of Interest

The authors declare no conflict of interest.

## Author Contributions

Q.S. conceived and supervised the project. R.T. carried out most of the experiments, including membrane fabrication, characterizations, and RFB tests. C.Y. synthesized the PIM‐EA‐TB polymer. A.W., B.P.D, L.P., Z.F., D.Z.L., J.X.L., and T.W. helped with experiments. A.A.F., M.F., and S.G. helped with the ellipsometry measurements. C.E.B. and K.E.J. contributed to the molecular simulations. P.A.A.K. and A.R.K. contributed to scientific discussions and the editing of the manuscript. Q.S. and R.T. wrote the manuscript with contributions from all authors. All authors discussed the results and commented on the manuscript at all stages.

## Supporting information

Supporting Information

## Data Availability

The data that support the findings of this study are available from the corresponding author upon reasonable request.
