# Supplementary material for: Thin Film Composite Membranes with Regulated Crossover and Water Migration for Long‐Life Aqueous Redox Flow Batteries
Source: Adv Sci (Weinh). 2023 May 13;10(20):2206888. doi: 10.1002/advs.202206888 (PMC10369228; doi:10.1002/advs.202206888)
Supplement: Supplementary file 1 — Supporting Information [file ADVS-10-2206888-s001.pdf]

ADVANCED SCIENCE
Open Access

Supporting Information

for *Adv. Sci.*, DOI 10.1002/advs.202206888

Thin Film Composite Membranes with Regulated Crossover and Water Migration for Long-Life Aqueous Redox Flow Batteries

*Rui Tan, Anqi Wang, Chunchun Ye, Jiaxi Li, Dezhi Liu, Barbara Primera Darwich, Luke Petit, Zhiyu Fan, Toby Wong, Alberto Alvarez-Fernandez, Mate Furedi, Stefan Guldin, Charlotte E. Breakwell, Peter A. A. Klusener, Anthony R. Kucernak, Kim E. Jelfs, Neil B. McKeown and Qilei Song**

Supplementary Materials for

Thin Film Composite Membranes with Regulated Crossover and Water Migration for Long-Life Aqueous Redox Flow Batteries

Rui Tan[1], Anqi Wang[1], Chunchun Ye[2], Jiaxi Li[1], Dezhi Liu[1], Barbara Primera Darwich[1], Luke Petit[1], Zhiyu Fan[1], Toby Wong[1], Alberto Alvarez-Fernandez[3], Mate Furedi[3], Stefan Guldin[3], Charlotte E. Breakwell[4], Peter A. A. Klusener[5], Anthony R. Kucernak[4], Kim E. Jelfs[4], Neil B. McKeown[2], Qilei Song[1]*

[1]Department of Chemical Engineering, Imperial College London, London SW7 2AZ, UK.
[2]EaStChem School of Chemistry, University of Edinburgh, Edinburgh, EH9 3FJ, UK.
[3]Department of Chemical Engineering, University College London, London WC1E 7JE, U.K
[4]Department of Chemistry, Molecular Sciences Research Hub, Imperial College London, London W12 0BZ, UK.
[5]Shell Global Solutions International B.V., Energy Transition Campus Amsterdam, Grasweg 31, 1031 HW Amsterdam, The Netherlands.

Email: q.song@imperial.ac.uk

Materials and Methods

Materials

Redox-active chemistries and supporting salts: Redox chemicals were purchased from Sigma-Aldrich and TCI, including potassium ferrocyanide ($K_4Fe(CN)_6$, >99%), 2,6-dihydroxy-anthraquinone (2,6-DHAQ, >90%), 4-hydroxy-tempo (4-OH-Tempo, >97%), methyl viologen dichloride hydrate (MV, >98%), anhydrous 1,1'-Bis[3-(trimethylammonio)propyl]-4,4'-bipyridinium tetrachloride (BTMAP-Vi, >98%), (Ferrocenylmethyl)trimethylammonium chloride (FcNCl, >95%), anhydrous zinc chloride ($ZnCl_2$, >99%), anhydrous potassium chloride (KCl, >99%), anhydrous sodium chloride (NaCl, >99%), anhydrous lithium chloride (>98%), anhydrous calcium chloride ($CaCl_2$, >97%), anhydrous magnesium chloride ($MgCl_2$, >98%), potassium hexacyanoferrate ($K_3Fe(CN)_6$, >99%), potassium hydroxide (anhydrous, >99%), sodium hydroxide (NaOH, >99%) and zinc plate. 2,6-DPPAQ was synthesized according to a reported method.[1]

Membranes: Nafion®212 (Dupont) membranes were purchased from Sigma-Aldrich. Nafion 212 membranes were treated in deionized water at 80°C for 30 min and immersed in 6 wt% hydrogen peroxide for 15 or 35 mins, subsequently stored in a 0.1 M KCl solution before use.

Methods

Fabrication of thin film composite (TFC) membranes. PIM-EA-TB polymer was synthesized following the previously reported protocol.[2] Obtained PIM-EA-TB polymer was dissolved in chloroform to produce PIM solutions with varied concentrations of 2.0, 4.0, 6.0 and 8.0 wt%. Such solutions were filtered through syringe filters (PTFE, 0.45 μm) or centrifuged to remove impurities. TFC membranes were fabricated by spin-coating dilute polymer solutions onto a porous polyacrylonitrile (PAN) substrate following previous protocol.[3] PIM solutions were dropped onto PAN supports by a spin-coating approach with a speed of 2000 rpm for 1 min. For the simplicity in AFM measurements, PIM solutions were coated onto silicon wafer supports and manually cracked before use.

Membrane characterization

Scanning electron microscopy (SEM) was performed using a Hitachi S5500 microscope to study the morphology of polymetric supports and TFC membranes. The thicknesses of the selective layers were determined by the cross-sectional SEM images. Before measurements, the samples were manually cracked in liquid nitrogen and fully dried in a vacuum oven at 80°C. The voltages of electron beams were adjusted in the range of 5~15 V in order to obtain the high resolution.

Atomic force microscopy (AFM) was carried out with a Bruker equipment (Bruker Scanasyst Dimension Icon AFM facility) using a peak force tapping mode in ambient air. AFM was used to prove the defect-free morphology and roughness of selective layers in TFC membranes.

Contact angle measurements was performed on a drop shape analyzer (KRÜSS Scientific Instrument) using deionized water or 1 M alkaline solution as testing solutions. The shape change of liquid drops was recorded for 30 s.

Pervaporation was carried out by sandwiching TFC membranes in a H-type cell with one side filled with deionized water while the other side was empty. The H-cell was stored in an oven at 70°C with 20% humidity, in which the part filled with water was completely sealed. Water molecules can pass through the membranes and evaporate from the empty side. Mass loss for the whole setup was recorded after certain time.

Dynamic vapour sorption (DVS) was performed on a DVS Endeavour gravimetric sorption analyser (Surface Measurement Systems) at 25 °C. Free standing PIM-EA-TB thin films were prepared by spin-coating PIM-EA-TB solutions onto glass slides, followed by the release of the thin films in deionized water. The collected films were dried in a vacuum oven at 110 °C for 12 h, and in situ dried under flowing dry air at room temperature for at least 24 h.

Ellipsometric porosimetry (EP) measurements were carried out on a Semilab SE-2000 variable angle spectroscopic ellipsometer (within the spectral range of 248 to 1653 nm) in a controlled humidity chamber. In order to obtain more reliable ellipsometric measurements, equivalent films to those previously presented in this work were prepared by spin-coating the polymeric solution onto silicon wafers. All ellipsometric data were analysed with Semilab SEA software, using the Tauc-Lorentz and Gauss dispersion laws for optical model fitting. During ellipsometric porosimetry measurements, ellipsometric spectra were recorded stepwise at 40 RH steps (in the range between 0.5%–100%) to obtain refractive index and thickness isotherms. The modified Kelvin equation was used to obtain pore size distribution (PSD) information as described previously.[4]

Pressure-driven permeation (nanofiltration) was carried out in a dead-end cell with water as the feed side at 30 bars. The water permeance was calculated based on the following equation:

$$J = \frac{V}{A \times t \times P} \quad \text{(S1)}$$

where V is the volume of permeated water; A is the effective area of the TFC membranes; t and P are the testing time and applied pressure, respectively.

Concentration-driven dialysis diffusion. Crossover of redox species was measured with a H cell, in which a TFC membrane was sandwiched in the middle, and 0.1M $K_4Fe(CN)_6$ and water were used as the feed side and permeate side, respectively. The concentration of Fe in permeated redox species was determined using ICP-OES. The permeance of $K_4Fe(CN)_6$ was calculated following Fick's first law:

$$J = \frac{V}{A}\left(\frac{\partial C}{\partial x}\right) \quad \text{(S2)}$$

where J is the permeance, V is the solution volume, A is the membrane effective area, and C and t are the concentration and diffusion time, respectively.

Electrochemical impedance spectroscopy (EIS) was used to measure the ohmic resistance of TFC membranes using the potentiostat mode (Biologic) with a voltage bias of 10 mV and a frequency range of 0.5 MHz-10Hz. TFC membranes were saturated with 1.0 M KCl or 0.1 M $K_4Fe(CN)_6$ and sandwiched between two block electrodes, i.e., stainless steel, in a coin-type cell. The ohmic resistance (R_{ohmic}) of membranes are the intersection points between EIS curves and the x-axis. The ionic conductivity for these membranes in the temperature range of room temperature to 70°C can be calculated according to the following equation:

$$\sigma = \frac{l}{A \times R_{ohmic}} \quad \text{(S3)}$$

where σ is the ionic conductivity, l is the membrane thickness, A is the effective area and R_{ohmic} is the membrane resistance. In the coin-type cells, the effective area is 2.0 cm^2. The overall thicknesses of the TFC membranes were determined by a micrometer; the thickness of the selective layer was tested by SEM. Arrhenius plots were derived using the ionic conductivity in the temperature range of room temperature to 70 °C. The Arrhenius equation was employed to linearly fit $ln(\sigma)$-$\frac{1}{T}$ plots to obtain activation energy following the equations below (S4-S5).

$$\sigma = \sigma_0 \exp\left(-\frac{E_a}{kT}\right) \quad \text{(S4)}$$

$$ln\sigma = ln\sigma_0 - \frac{E_a}{kT} \quad \text{(S5)}$$

Where T is the temperature, σ_0 is the pre-exponential factor, E_a and k are the activation energy and Boltzmann constant.

The resistance of assembled flow batteries was measured with the same protocol. Additionally, EIS was used to investigate the ohmic resistance of polymeric supports hydrolyzed in 1.0 M KOH water/ethanol (v:v=1:1) solution under various pre-treatment time. PAN supports hydrolyzed at above 4 h lost their mechanical properties and became very soft and brittle.

Cyclic voltammetry was employed to measure the redox reactions of active species using a three-electrode system on the Biologic electrochemical workstation. The three-electrode system was built up with a glassy carbon working electrode, a Pt counter electrode and a Ag/AgCl reference electrode (3M KCl) and a diluted redox-species solution (10 mM). The redox processes of Zn^{2+}-ion, MV, TEMPO, $K_4Fe(CN)_6$, BTMAP-Vi, FcNCl and 2,6-DPPAQ were determined using the CV method with a scanning rate of 10 mV/s.

Transference number was measured using a H-type cell where a target membrane was sandwiched between 1.0 M and 0.1 M salt solutions. Ag/AgCl reference electrodes (3.0 M KCl) were placed in each half cell to form two electrode system. I-V scanning was carried out to derive the zero-current voltage (V_0), and the transference number was calculated based on the following equation[5]:

$$V_0 = \left(\frac{t_+}{Z_+} - \frac{t_-}{Z_-}\right)\left(\frac{kT}{e}\right)\ln\left(\Delta C \times \frac{\gamma_{high}}{\gamma_{low}}\right) \quad \text{(S6)}$$

where t_+ and t_- are the transference number for cations and anions; Z_+ and Z_- are the valence value for the ions; γ_{high} and γ_{low} are the mean activity coefficients for the salt solutions with high and low concentrations, respectively. The mean activity coefficients were calculated based on the previous

reports[6]. The ratio of cation mobility (μ_+) to anion mobility (μ_-) can be calculated based on the equation[7]:

$$\frac{\mu_+}{\mu_-} = -\left(\frac{Z_+}{Z_-}\right)\left(\frac{ln\Delta - Z_- FV_0/RT}{ln\Delta - Z_+ FV_0/RT}\right) \quad (S7)$$

The ionic conductivity for certain cations or anions (i) was calculated based on the following equation:

$$\sigma_i = \frac{\sigma \times t_i}{z_i^2} \quad (S8)$$

where σ is the conductivity of the salt and t_i is the transference number of ions (i). Subsequently, we could obtain the ion selectivity of cations to anions following the equation:

$$S = \frac{\sigma_+}{\sigma_-} \quad (S9)$$

Flow battery setup and performance test

Electrolyte preparation. Zn^{2+}-ion||TEMPO: 0.136 g $ZnCl_2$ and 0.172 g TEMPO were dissolved in 1 M KCl solutions (10 mL) as the anolyte and catholyte; Zn^{2+}-ion|| $K_4Fe(CN)_6$: 0.42 g $K_4Fe(CN)_6$ was dissolved in a 1 M KCl solution (10 mL) as the catholyte; The preparation of anolytes and catholytes for MV||TEMPO and BTMAP-Vi||FcNCl followed the same protocol. 2,6-DPPAQ||$K_4Fe(CN)_6$: firstly, a 1M KCl solution was mixed with trace amount of KOH solution to afford a pH=9 solution; 0.1 M 2,6-DPPAQ and $K_4Fe(CN)_6$ were prepared within the above solution to form an anolyte and a catholyte, respectively.

Battery assembly. Cell hardware from Scribner Associates was used to assemble a flow cell with two POCO graphite flow graphite bipolar plates and carbon papers that were pre-baked at 400°C for 24 h in the air. Carbon paper (Sigracet SGL 10AA) was purchased from Fuel Cell Tech and cut into 7 cm^2 sheets before use. Nafion 212 and TFC membranes served as the ion conductive and molecular selective separators assembled in the middle. A Cole-Parmer Micropump gear pump was used to flow the electrolytes in and out of the reaction cells with a flow rate of 100 mL min^{-1}. Before assembly, membranes were stored in near-/ neural solutions.

Cycling and power density tests. The cut-off potentials for Zn^{2+}-ion||TEMPO, MV||TEMPO, Zn^{2+}-ion|| $K_4Fe(CN)_6$, BTMAP-Vi||FcNCl and 2,6-DPPAQ|| $K_4Fe(CN)_6$ redox pairs were 0.8-1.9, 0.6-1.7, 0.8-1.7, 0.5-1.5 and 0.5-1.5 V. The cycling performance of RFBs was evaluated at 20 and 80 mA cm^{-2} in an argon-filled glovebox. To accurately obtain the capacity retention, RFBs were fully discharged at a low current density of 10 mA cm^{-2}. Polarization curve measurements were performed with a chronopotentiometry method from 0 to 6000 mA as an interval of 200 mA. In each polarization step, a constant current was applied for RFBs until the potential reached the steady state, after which the battery was charged back using the same current before proceeding to next step of polarization. State of charge (SOC) for these RFBs were achieved by charging to certain amount of capacity. Energy efficiency and coulombic efficiency can be directly collected from the Biologic electrochemical workstation. Capacity of these RFBs can be normalized by the discharge capacity at 1st cycle. CV was used to test the redox reactions of cycled anolytes and catholytes to estimate the practical crossover in the operational RFBs with a scanning rate of 10 mV/s.

RFBs with concentrated 2,6-DPPAQ|| $K_4Fe(CN)_6$ redox species were assembled with a PIM-EA-TB-4.0 membrane, 0.8 M $Fe(CN)_6^{4-}$ catholyte and 0.4M 2,6-DPPAQ anolyte. Catholyte was prepared by dissolving 2.6 mmol $K_4Fe(CN)_6$ and 2.6mmol $Na_4Fe(CN)_6$ in 6.5 mL mixed supporting electrolytes of 1.0 M KCl and 1.0 M NaCl. Anolyte was prepared by adding 2.6 mmol 2,6-DPPAQ in 6.5 mL mixed electrolyte of 0.8 M KOH and 0.8 M NaOH (for the deprotonation of phosphate groups in 2,6-

DPPAQ), and 1.8 M KCl and 1.8 M NaCl. The cation concentrations are equivalent in both electrolytes. A trace amount of KOH solution was added to the above solutions to adjust the pH to 9.0. To better evaluate the applicability of these batteries, RFBs with concentrated redox species were tested in the open air. The established batteries were tested at 20 mA cm^{-2} by limiting the charging time to 1 hour to evaluate the electrolyte migration change and cycling performance.

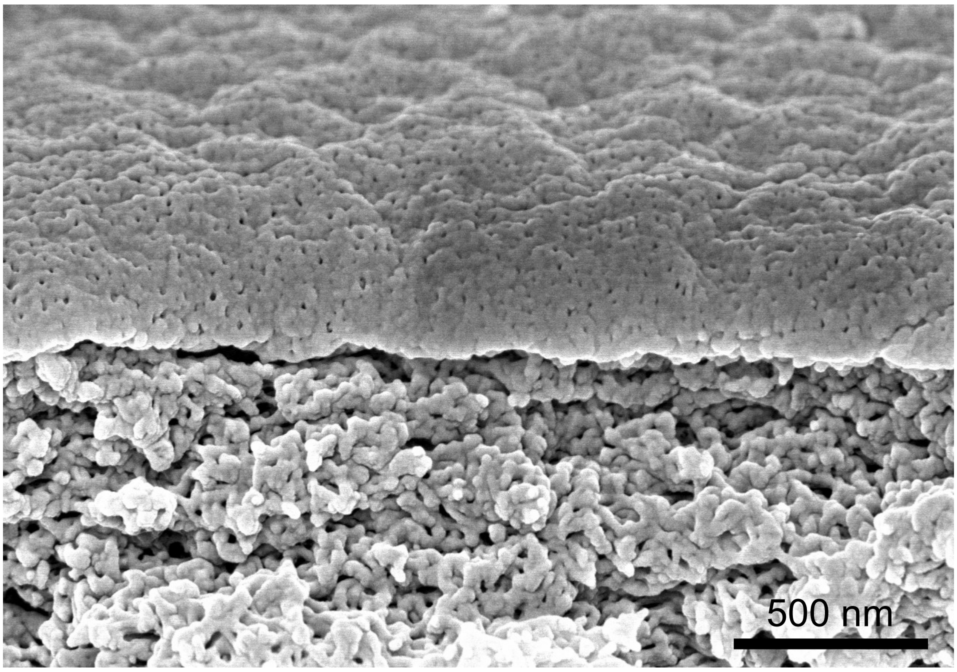

Figure S1. Cross-sectional morphology of a porous PAN support.

Table S1|Surface roughness and selective-layer thickness.

PIM-EA-TB concentration (wt%)	R_q (nm)	R_a (nm)	AFM thickness (nm)
2.0	0.468	0.345	374.2
4.0	0.691	0.547	929.9
6.0	1.29	1.03	N/A[a]
8.0	1.47	0.752	N/A[a]

[a] The thicknesses of selective layers for TFC membranes using 6.0 and 8.0 wt% are over the testing range of AFM.

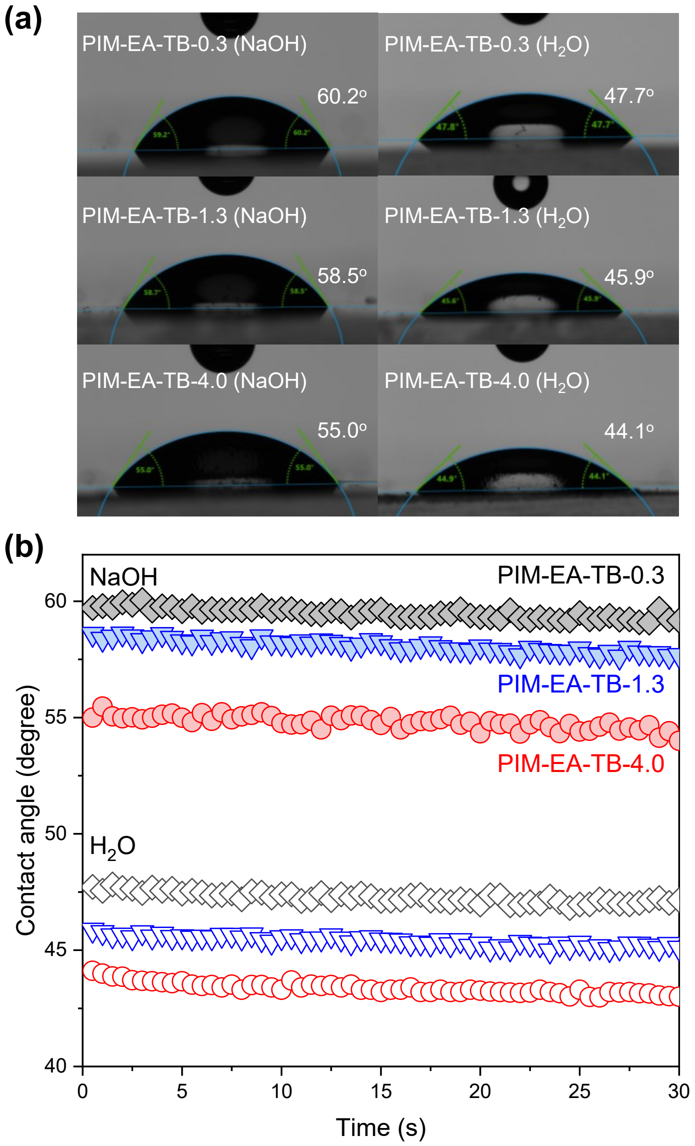

Figure S2. Membrane hydrophilicity. (a) Contact angles of PIM-EA-TB-0.3, PIM-EA-TB-1.3 and PIM-EA-TB-4.0 for water and a 1 M NaOH solution. (b) Contact angle change of PIM-EA-TB-0.3, PIM-EA-TB-1.3 and PIM-EA-TB-4.0 over 30 s.

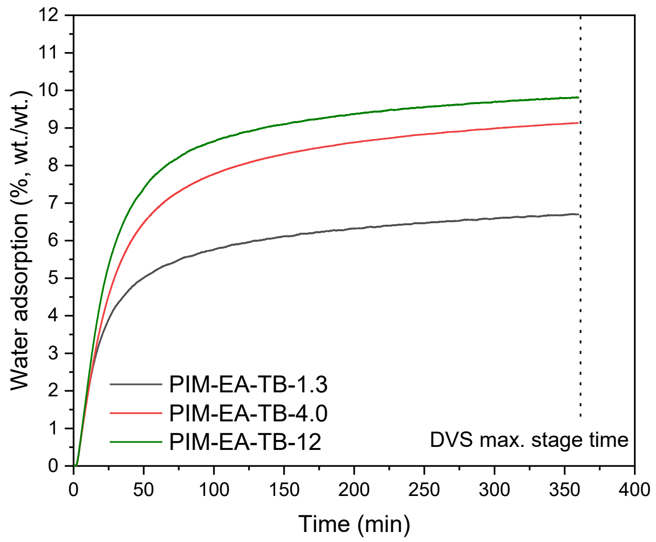

Figure S3. Dynamic vapor sorption profiles of PIM-EA-TB-1.3, PIM-EA-TB-4.0 and PIM-EA-TB-12 for 80% RH to 90% RH. For better comparison, these profiles were normalized by the water adsorption values at 80% RH. We didn't include PIM-EA-TB-0.3 in this test as its mass is very limited, for which the DVS results are not precise.

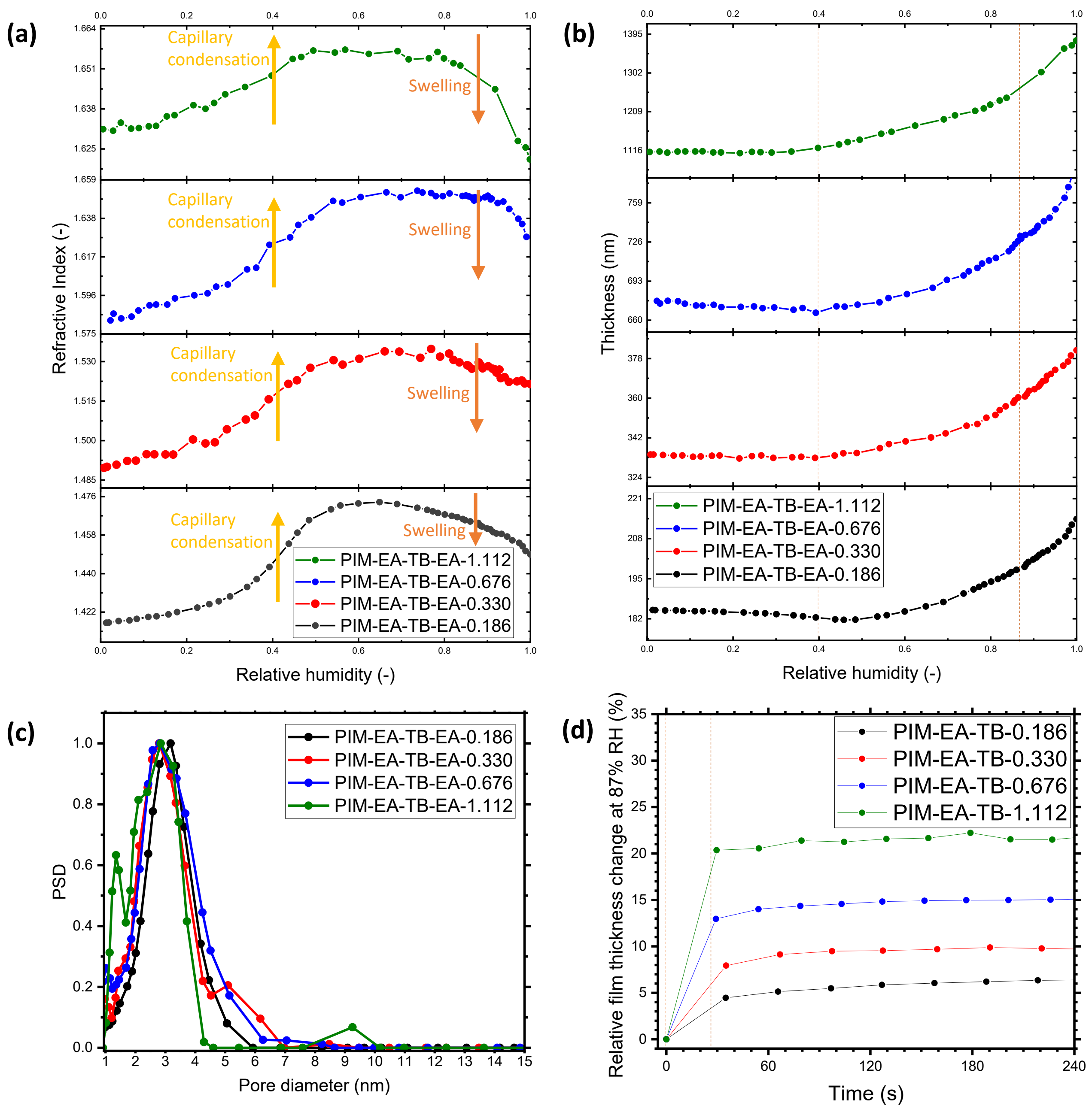

Figure S4. Ellipsometry tests. (a) Refractive index isotherms of PIM-EA-TB thin films at 0.5-100% RH water adsorption sequence (with 20 s wait time at each step). The capillary condensation and swelling have opposite effects on the refractive index, as marked in Fig. S4a. (b) Thickness change of PIM-EA-TB thin films during the same sequence. (c) Derived pore size distribution calculated by considering the capillary condensation. (d) In-situ thickness change monitoring in time during a 40-87% one-step RH increase and subsequent constant 87% RH environment (normalized to thickness at 40% RH).The fitted specific surface areas for PIM-EA-TB-0.186, PIM-EA-TB-0.330, PIM-EA-TB-0.676 and PIM-EA-TB-1.112 are 557, 404, 580 and 318 m^2/cm^3, respectively.

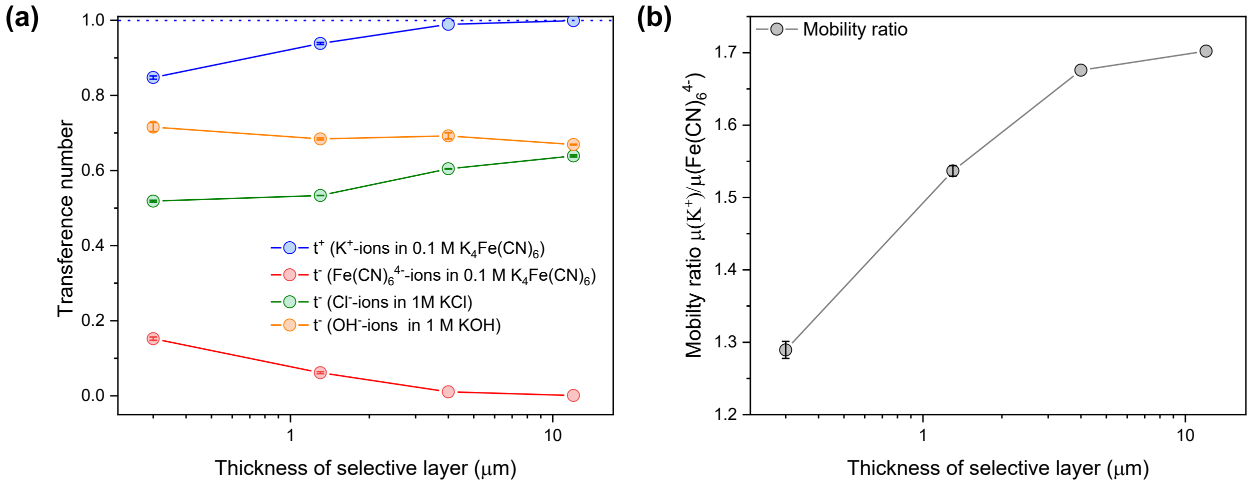

Figure S5. Transference number and mobility tests. (a) Transference number of various ions in PIM-EA-TB with varied thicknesses. (b) Mobility ratio of K^+ to $Fe(CN)_6^{4-}$ in PIM-EA-TB with varied thicknesses.

Table S2|Transference number of various ions in PIM-EA-TB with varied thicknesses.

PIM-EA-TB thickness (μm)	0.1 M|0.01 M $K_4Fe(CN)_6$			1 M|0.1 M KOH			1 M|0.1 M KCl		
	OCV (mV)	t^+	t^-	OCV (mV)	t^+	t^-	OCV (mV)	t^+	t^-
0.3	32.5 (±0.40)	0.847	0.143	-24.8 (±1.4)	0.284	0.716	-2.04 (±0.26)	0.481	0.519
1.3	40.9 (±0.45)	0.938	0.062	-21.2 (±0.31)	0.316	0.684	-3.67 (±0.04)	0.467	0.533
4.0	45.7	0.989	0.011	-22.2 (±0.92)	0.307	0.693	-11.4 (±0.06)	0.395	0.605
12	46.6	0.999	0.001	-19.4 (±0.15)	0.331	0.669	-15.2 (±0.31)	0.361	0.639

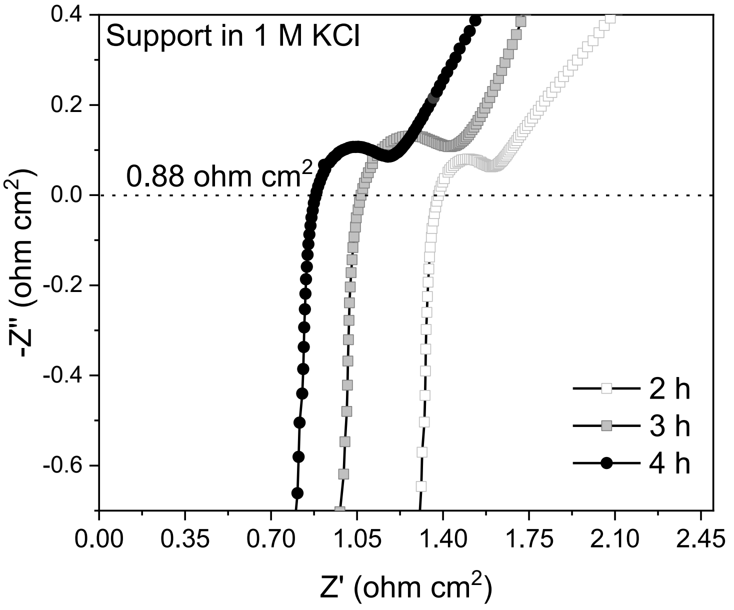

Figure S6. Nyquist plot of hydrolyzed PAN substrates for 2.0, 3.0 and 4.0 h. The PAN support hydrolyzed for longer than 4 h became very soft and brittle, which failed to work as a robust support for PIM-EA-TB thin films.

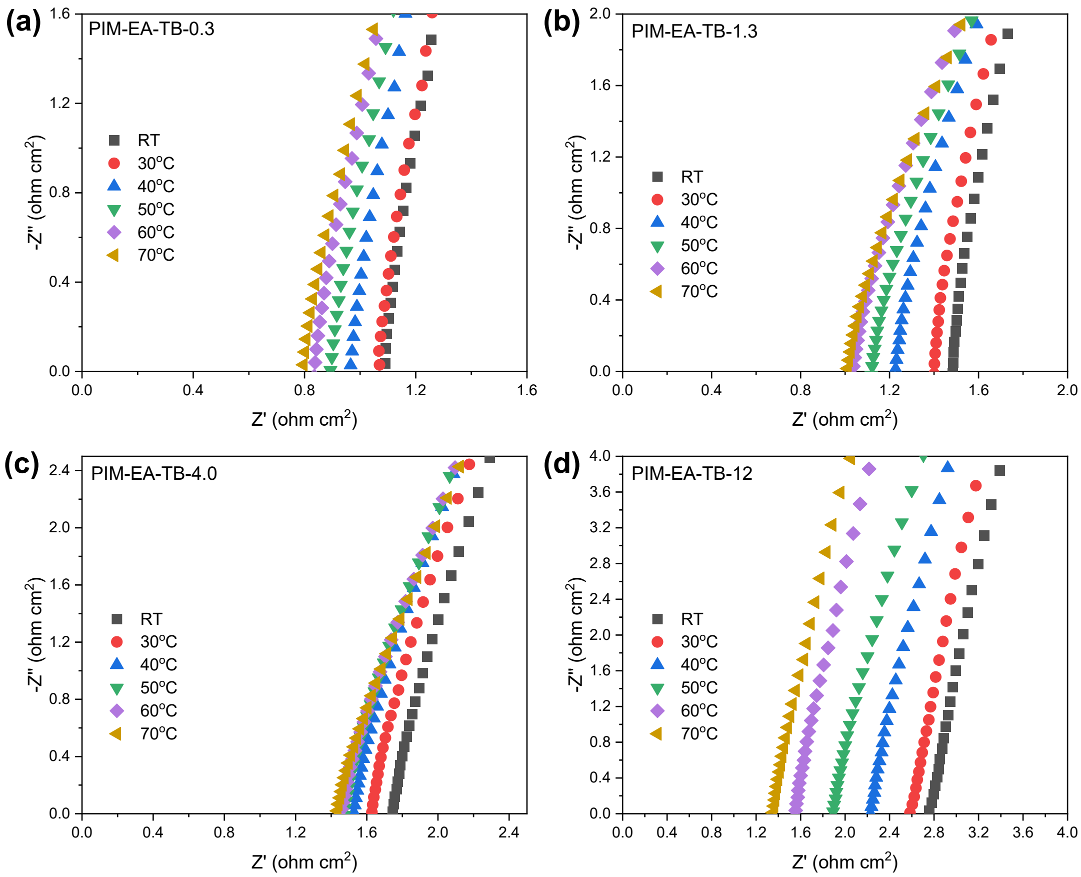

Figure S7. Electrochemical impedance spectroscopy (EIS). EIS Nyquist plots of (a) PIM-EA-TB-0.3, (b) PIM-EA-TB-1.3, (c) PIM-EA-TB-4.0 and (d) PIM-EA-TB-12 in 1 M KCl in the temperature range of RT to 70°C

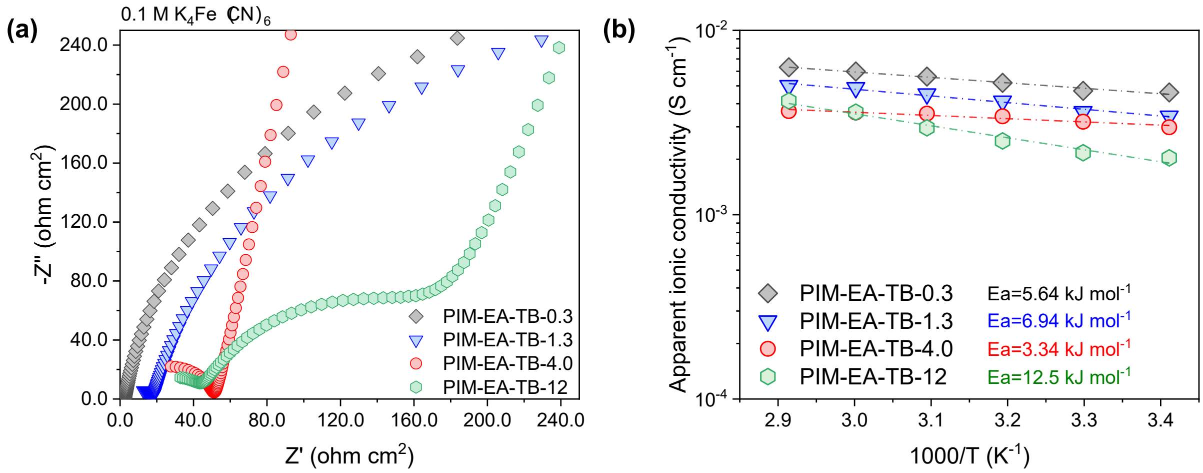

Figure S8. (a) Ion transport resistance of PIM-EA-TB TFC membranes in 0.1 M $K_4Fe(CN)_6$. (b) Arrhenius plots for the TFC composite membranes in 1 M KCl solutions. Cross-membrane ionic conductivity refers to the overall ion-conducting performance of selective layers and supports.

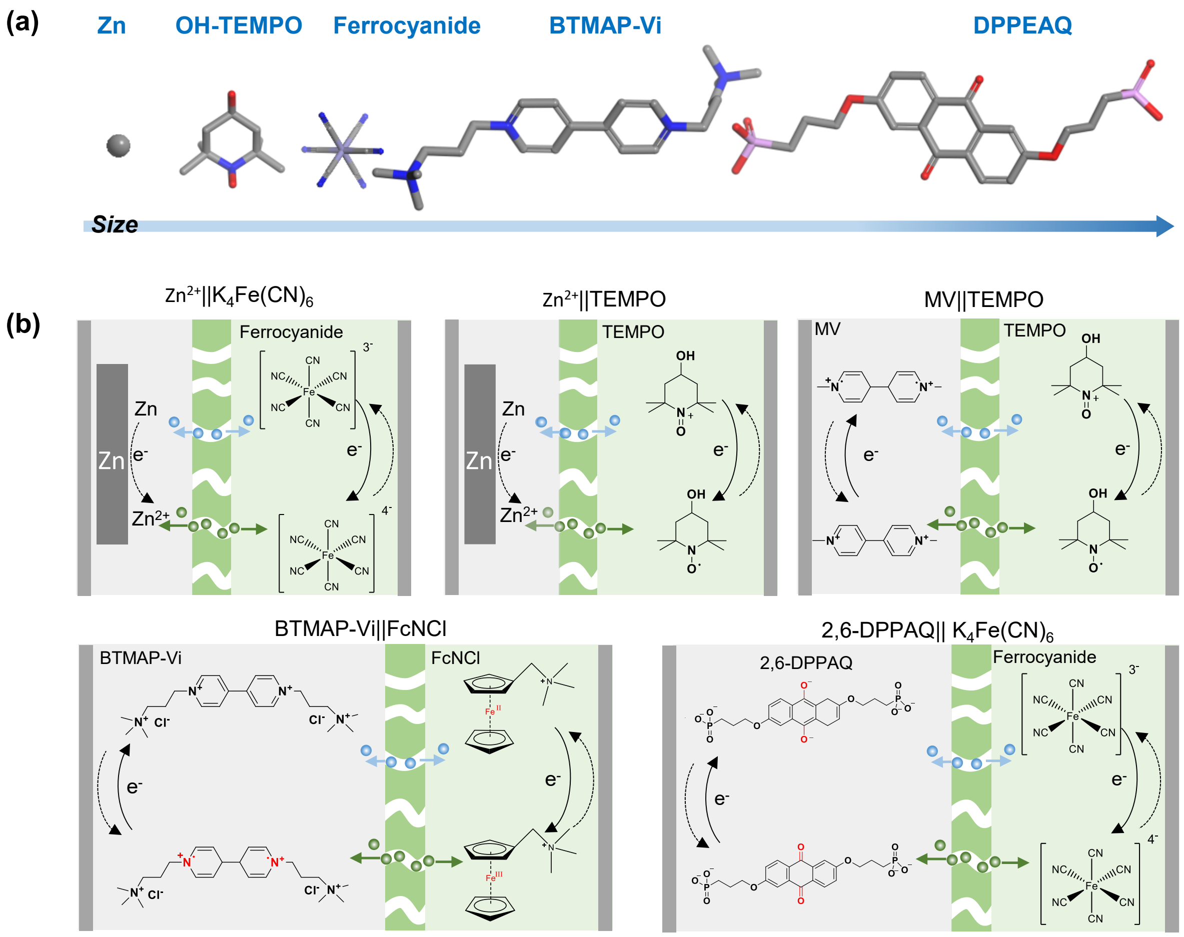

Figure S9. Size and redox potential of active chemistries. (a) Molecular size comparison of selected redox chemicals. (b) Schematic illustration showing five pairs of redox chemistries and their redox reaction mechanisms.

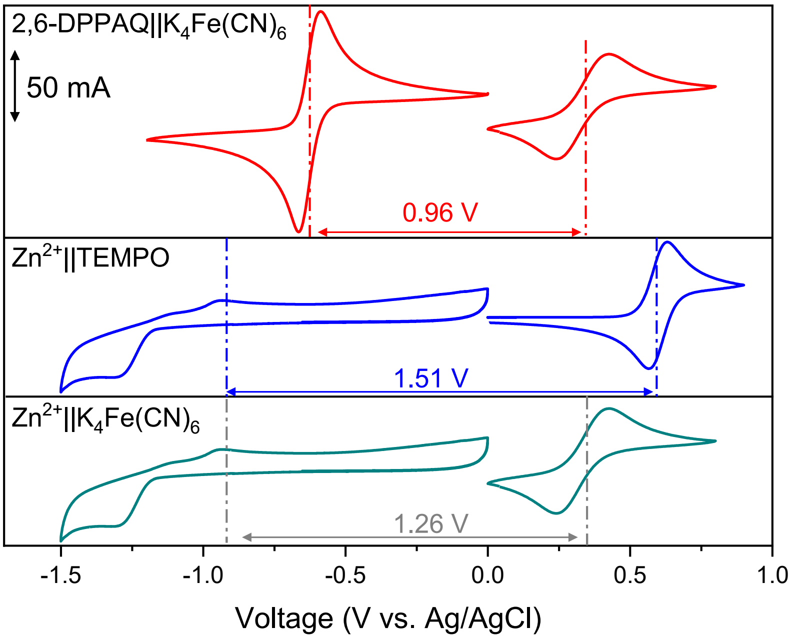

Figure S10. Cyclic voltammetry curves of three redox couples, 2,6-DPPAQ||$K_4Fe(CN)_6$, Zn^{2+}-ion||TEMPO and Z^{2+}-ion|| $K_4Fe(CN)_6$. Theoretical potentials of these redox couples were marked in this plot.

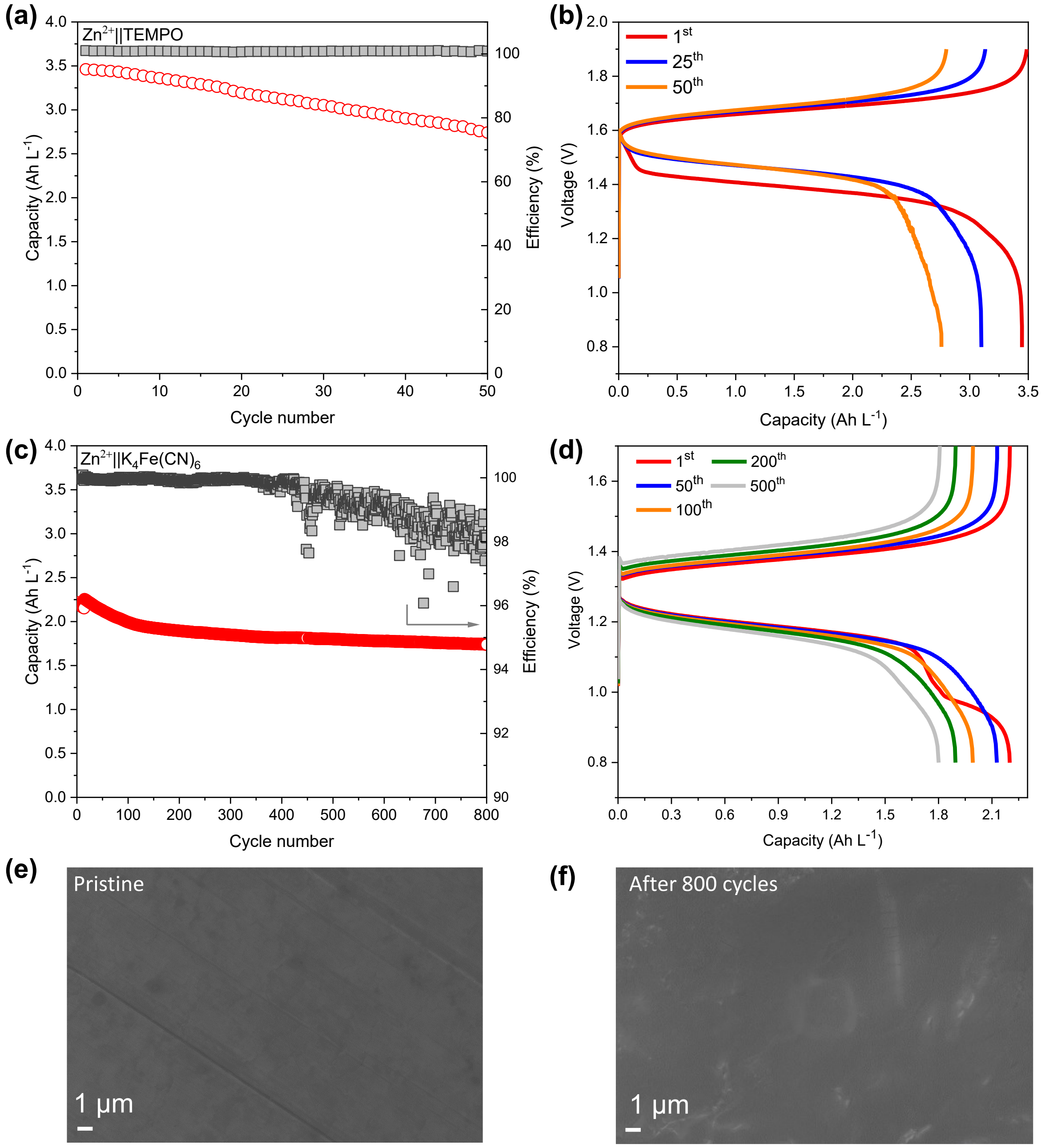

Figure S11. Zn-based RFB performance with a PIM-EA-TB-0.3 membrane. (a) Cycling performance and (b) typical dis-/ charging curves of a Zn^{2+}-ion||TEMPO battery. Inset shows the fast permeation of small-sized TEMPO through a membrane (c) Cycling performance and (d) typical dis-/ charging curves of a Zn^{2+}-ion||$K_4Fe(CN)_6$ battery. Insets shows that the large-sized $K_4Fe(CN)_6$ can be blocked by a PIM-EA-TB-0.3 membrane. (e-f) SEM images showing the morphologies of zinc metal before and after 800 cycles.

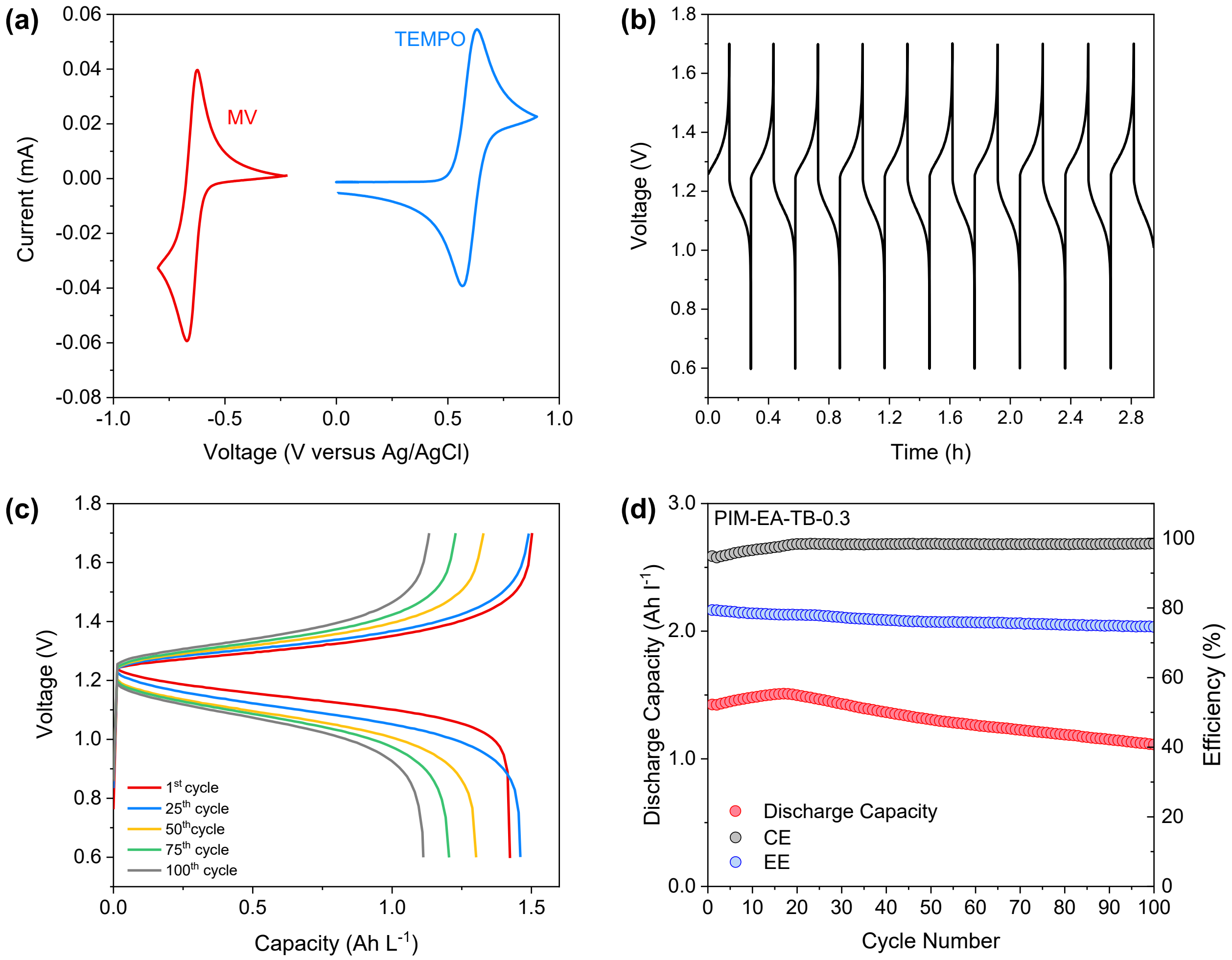

Figure S12. MV||TEMPO RFB performance with a PIM-EA-TB-0.3 membrane. (a) CV plots of MV and TEMPO redox species. (b) Cycling curves against running time. (c) Typical dis-/ charging profiles of a MV||TEMPO RFB. (d) Cycling performance of a MV||TEMPO RFB.

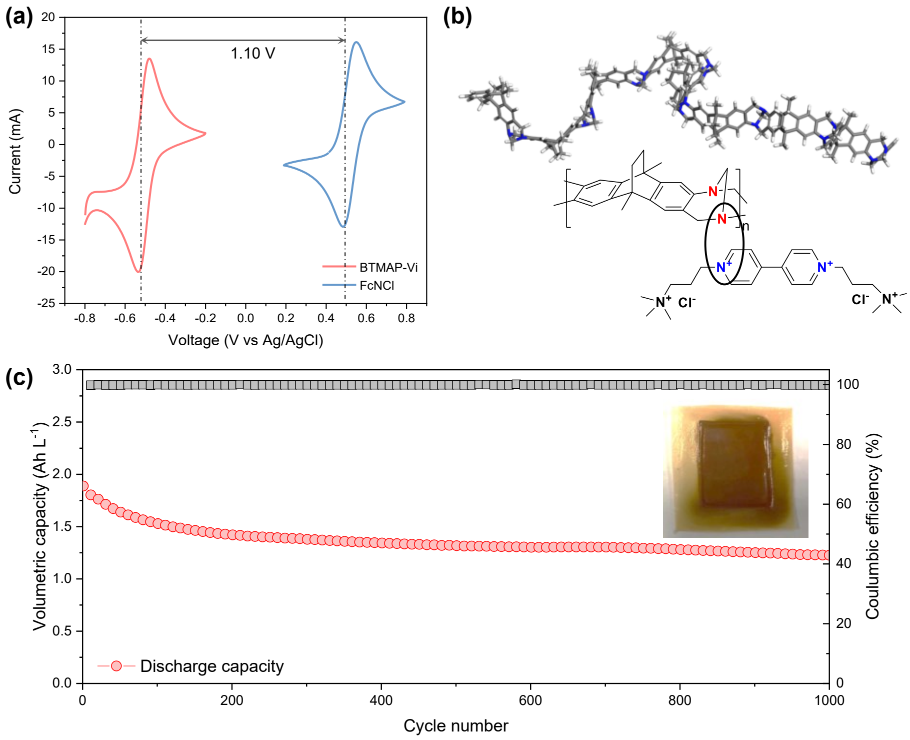

Figure S13. BTMAP-Vi||FcNCl RFB performance with a PIM-EA-TB-0.3 membrane. (a) CV plots of BTMAP-Vi and FcNCl redox species. (b) Polymer structure for PIM-EA-TB. Tröger's base units might have interaction with positively charged species and cause fouling of the membrane. (c) Cycling performance of a BTMAP-Vi||FcNCl RFB. Inset shows the contaminated PIM-EA-TB-0.3 membrane.

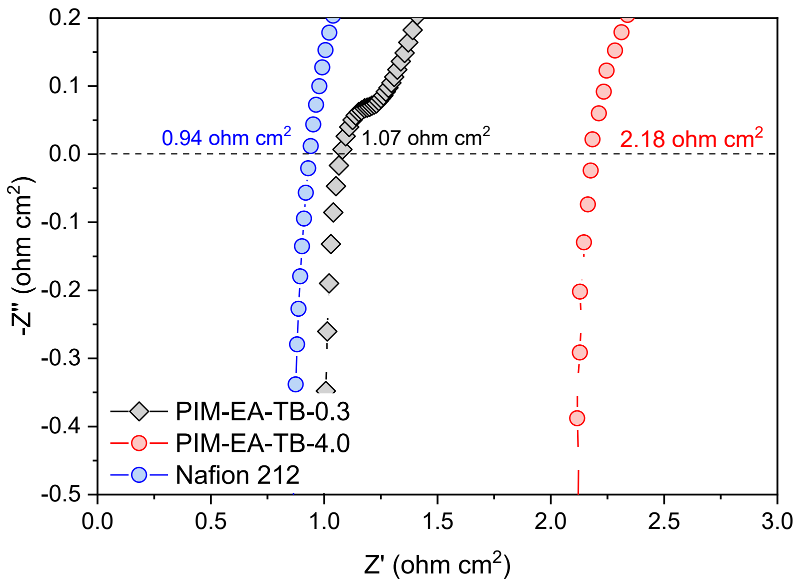

Figure S14. Nyquist plots of PIM-EA-TB-0.3, PIM-EA-TB-4.0 and Nafion 212 in redox flow batteries with 0 SOC.

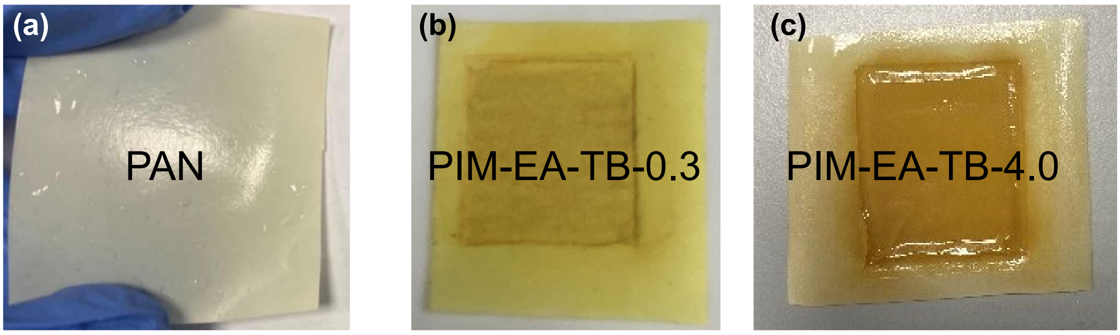

Figure S15. Photos of (a) a polymeric PAN support (b) a cycled PIM-EA-TB TFC membrane with 0.3-µm selective layer and (c) a cycled PIM-EA-TB TFC membrane with 4.0-µm selective layer.

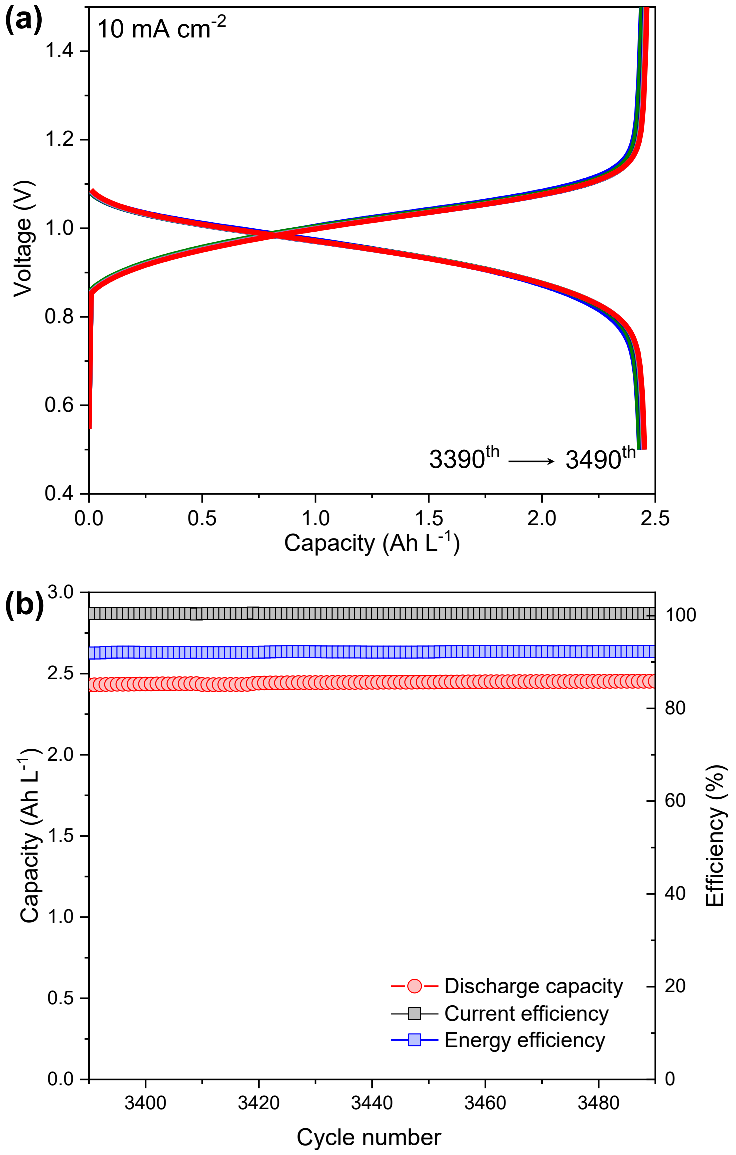

Figure S16. Cycling performance of PIM-EA-TB-4.0 at a low current. (a) Dis-/ charging profiles of the 2,6-DPPAQ||$K_4Fe(CN)_6$ RFB with a PIM-EA-TB-4.0 membrane over 100 cycles. (b) Discharge capacity, energy efficiency and current efficiency for the 2,6-DPPAQ||$K_4Fe(CN)_6$ RFB with a PIM-EA-TB-4.0 membrane over 100 cycles.

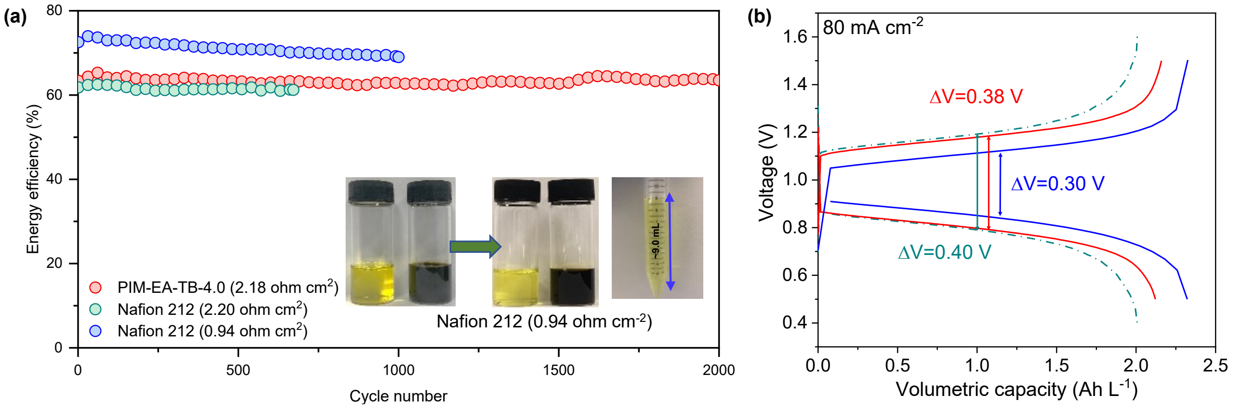

Figure S17. Long-cycling performance of Nafion 212 and PIM-EA-TB-4.0. (a) Energy efficiency of RFBs with Nafion 212 (0.94 ohm cm^2 and 2.20 ohm cm^2) and PIM-EA-TB-4.0. (b) Typical charging-discharging profiles of RFBs with Nafion 212 (0.94 ohm cm^2 and 2.20 ohm cm^2) and PIM-EA-TB-4.0 membranes. The resistance of Nafion 212 will be determined by the pretreating methods. Inset of Fig.S17a shows the water migration of a RFB with a Nafion 212 membrane (0.94 Ω cm^2).

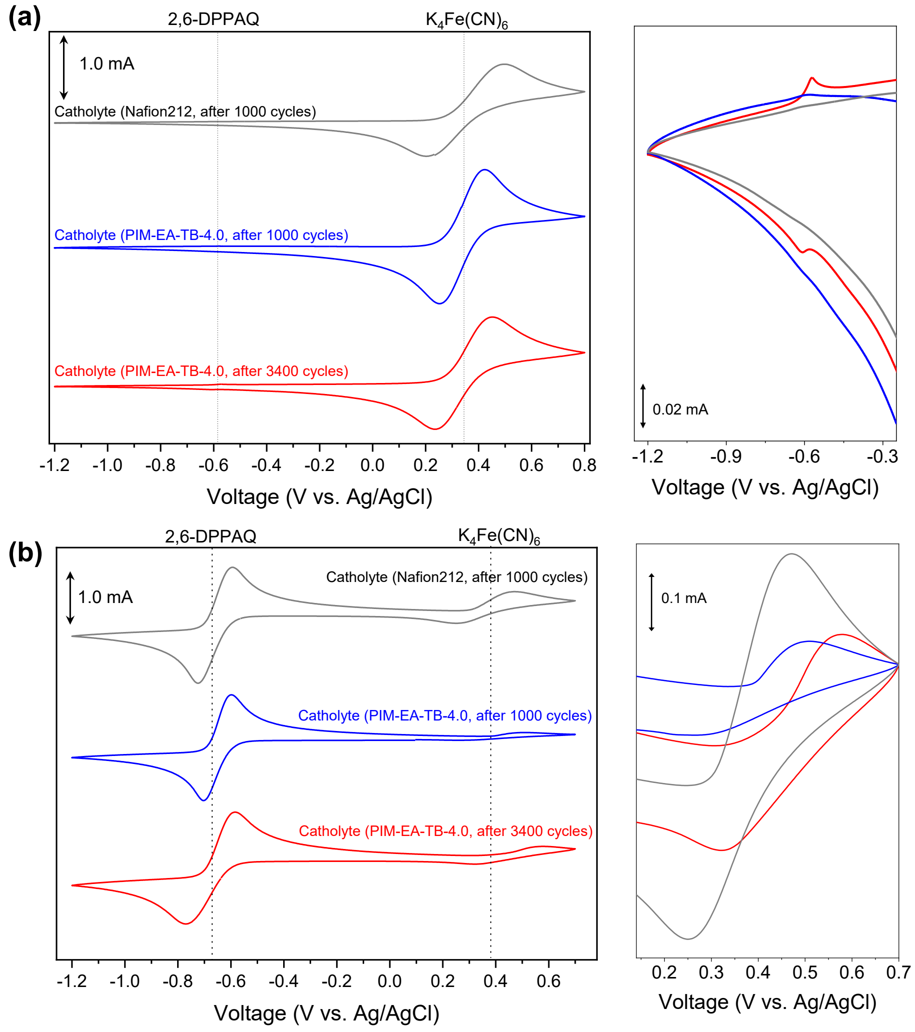

Figure S18. Cyclic voltammetry curves of cycled anolytes and catholytes. (a) CV curves of catholytes from the 2,6-DPPAQ||$K_4Fe(CN)_6$ RFBs with Nafion 212 and PIM-EA-TB-4.0 membranes after certain cycles. (b) CV curves of anolytes from the 2,6-DPPAQ||$K_4Fe(CN)_6$ RFBs with Nafion 212 and PIM-EA-TB-4.0 membranes after certain cycles.

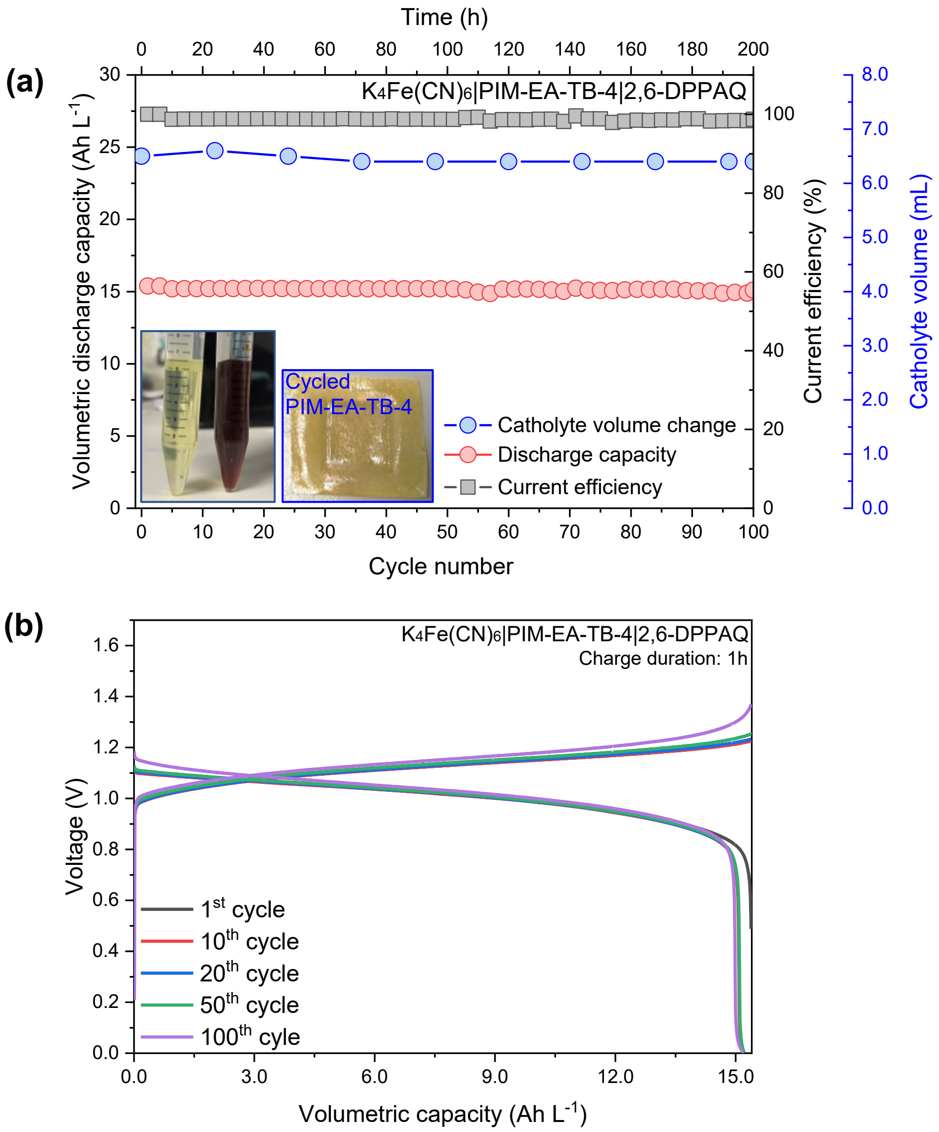

Figure S19. Cycling performance of PIM-EA-TB-4.0 in a battery with concentrated 2,6-DPPAQ||$K_4Fe(CN)_6$ redox species in the open air. (a) Cycling performance and volume change of this battery over 200 hours at 20 mA cm^{-2}. (b) Typical charging-discharging profiles.

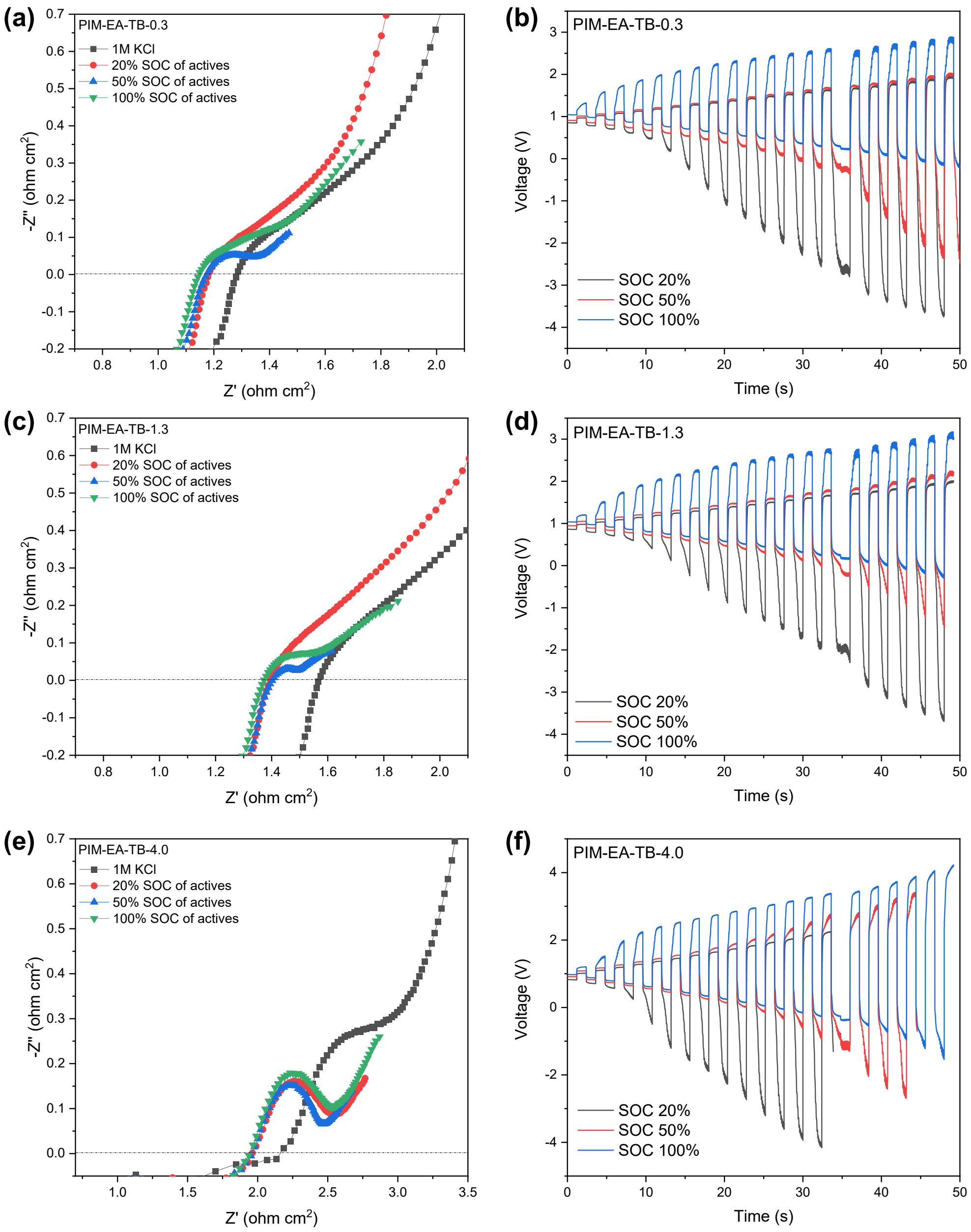

Figure S20. Polarization measurement. EIS measurements and polarization tests for (a-b) PIM-EA-TB-0.3, (c-d) PIM-EA-TB-1.3 and (e-f) PIM-EA-TB-4.0 with 0.1 M 2,6-DPPAQ and 0.1 M $K_4Fe(CN)_6$ redox species at 20%, 50% and 100% SOC.

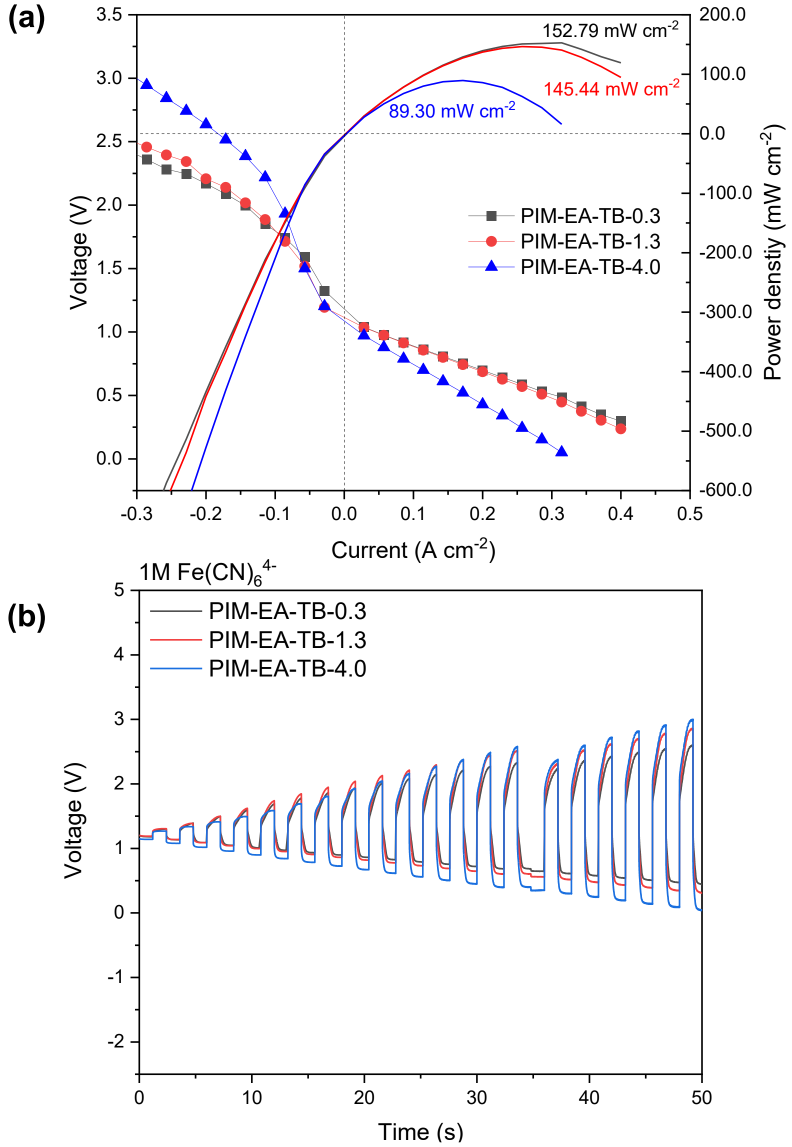

Figure S21. Powder density. (a) Comparison of the power densities of PIM-EA-TB-0.3, PIM-EA-TB-1.3 and PIM-EA-TB-4.0 with 0.1 M 2,6-DPPAQ and 0.1 M $K_4Fe(CN)_6$ redox species at 100% SOC. (b) Comparison of the polarization curves of PIM-EA-TB-0.3, PIM-EA-TB-1.3 and PIM-EA-TB-4.0 with 1.0 M 2,6-DPPAQ and 1.0 M $K_4Fe(CN)_6$ redox species at 100% SOC.

Table S3|Cost analysis of PIM-EA-TB production. The yield of intermediates/products are reported in brackets.

**Raw Materials**	**Price in £ & vendors**	**Intermediates/products**
Magnesium turnings<br>(2 equiv., 10.0 g)	£ 0.04/g<br>Fluorochem	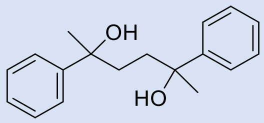<br>**Compound 1** (61 %)
Bromobenzene<br>(2 equiv., 64.6 g)	£ 0.03/g<br>Fluorochem	
2,5-hexanedione<br>(1 equiv., 24.0 mL)	£ 0.30/mL<br>Fluorochem	
Compound 1<br>(1 equiv., 34.1 g)		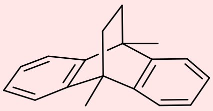<br>**Compound 2** (30 %)
Aluminium trichloride<br>(1 equiv., 16.7 g)	£ 0.03/g<br>VWR Chemicals	
Compound 2<br>(1 equiv., 13.5 g)		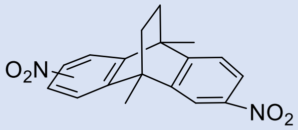<br><br>**Compound 3** (88 %)
Potassium nitrate<br>(2.1 equiv., 12.2 g)	£ 0.004/g<br>VWR Chemicals	
Trifluoroacetic anhydride<br>(7 equiv., 83.9 g)	£ 0.05/g<br>Fluorochem	
Compound 3<br>(1 equiv., 26.5 g)		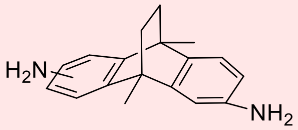<br><br>**Compound 4** (95 %)
Raney nickel<br>(1.0 wt%, ~0.265 g)	£ 0.34/g<br>Merck	
Hydrazine monohydrate<br>(20 equiv., 85.1 g)	£ 0.28/g<br>VWR Chemicals	
Compound 4<br>(1 equiv., 20.2 g)		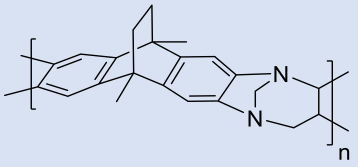<br><br>**PIM-EA-TB** (76 %)
Dimethoxymethane<br>(1 equiv., 33.5 mL)	£ 0.009/mL<br>Merck	
Trifluoroacetic acid<br>(87.8 g)	£ 0.03/g<br>Apollo Scientific	
Total cost: £ 41.1	Yield: 17.9 g	**Price: £ 2.3/g**

The cost analysis method refers to our previously reported work.[8] The PIM-EA-TB cost analysis was estimated according to the cost of synthesis ingredients, reagents and catalysts (cost data were obtained through vendors' websites on 05/09/2022). The final cost is £ 2.3/g for PIM-EA-TB polymer, £ 9.2/m^2 (4 µm thick) and £ 0.7/m^2 (0.3 µm thick) for PIM-EA-TB membranes, respectively. Notably, above simple estimation does not take the manufacturing and operational costs, *etc.* into account. We envision that the upscaled PIM-EA-TB synthesis and membrane manufacturing will further reduce the production cost.

Estimation of key properties for membranes

To better compare the key properties of membranes, we qualitatively ranked each performance on a scale from 1 to 5 (5: excellent; 1: bad). The rankings of ionic conductivity, the ability to limit redox-species crossover and cycling performance were directly evaluated based on the experimental values (Table S4). The cost of TFC membranes can be simply calculated based on the capital cost of ingredients without considering the synthesis and manufacturing costs (Table S3). PIM-EA-TB TFC membranes can be easily processed into a large size with the benefits of solution-processable properties, while the process of Nafion membranes is notoriously sophisticated alongside the generation of toxic byproducts perfluoroalkyl and polyfluoroalkyl substances.

Table S4|The key parameters for the ranking of properties of PIM-EA-TB TFC and Nafion membranes.

Parameters	PIM-EA-TB-0.3	PIM-EA-TB-4.0	Nafion 212
Ionic conductivity	5 (4.5 mS/cm in 1M KCl) [a]	4 (3.0 mS/cm in 1M KCl) [a]	5 (5.4 mS/cm in 1M KCl) [a]
Cost-effectiveness	5 (£0.7/m^2)	4 (£9.2/m^2)	1 (£492.3/m^2)
Processability	5	5	2
Safety	4	4	2
Crossover limitation ability	1 (25.8 mmol m^{-2} h^{-1}) [b]	4 (0.19 mmol m^{-2} h^{-1}) [b]	2 (11.7 mmol m^{-2} h^{-1}) [b]
Cycling performance in RFBs	3 (0.0074% per cycle) [c]	4 (0.0024% per cycle) [c]	1 (0.017% per cycle) [c]

[a] Values are the ionic conductivity of membranes in 1M KCl solutions.
[b] Values are the diffusion rates of redox species through membranes.
[c] Decay rates were derived from the cycling stability of near neutral RFBs with membranes.

References

[1] Y. Ji, M.-A. Goulet, D. A. Pollack, D. G. Kwabi, S. Jin, D. De Porcellinis, E. F. Kerr, R. G. Gordon, M. J. Aziz, *Adv. Energy Mater.* **2019**, 9, 1900039.

[2] M. Carta, R. Malpass-Evans, M. Croad, Y. Rogan, J. C. Jansen, P. Bernardo, F. Bazzarelli, N. B. McKeown, *Science* **2013**, 339, 303.

[3] R. Tan, A. Wang, R. Malpass-Evans, R. Williams, E. W. Zhao, T. Liu, C. Ye, X. Zhou, B. P. Darwich, Z. Fan, L. Turcani, E. Jackson, L. Chen, S. Y. Chong, T. Li, K. E. Jelfs, A. I. Cooper, N. P. Brandon, C. P. Grey, N. B. McKeown, Q. Song, *Nat. Mater.* **2020**, 19, 195.

[4] a) C. Boissiere, D. Grosso, S. Lepoutre, L. Nicole, A. B. Bruneau, C. Sanchez, *Langmuir* **2005**, 21, 12362; b) A. Alvarez-Fernandez, B. Reid, M. J. Fornerod, A. Taylor, G. Divitini, S. Guldin, *ACS applied materials & interfaces* **2020**, 12, 5195.

[5] Q. Dai, F. Xing, X. Liu, D. Shi, C. Deng, Z. Zhao, X. Li, *Energy & Environmental Science* **2022**, 15, 1594.

[6] a) J. Kielland, *Journal of the American Chemical Society* **1937**, 59, 1675; b) G. N. Lewis, M. Randall, *Journal of the American Chemical Society* **1921**, 43, 1112.

[7] J. Lu, H. Zhang, J. Hou, X. Li, X. Hu, Y. Hu, C. D. Easton, Q. Li, C. Sun, A. W. Thornton, *Nature materials* **2020**, 19, 767.

[8] C. Ye, A. Wang, C. Breakwell, R. Tan, C. Grazia Bezzu, E. Hunter-Sellars, D. R. Williams, N. P. Brandon, P. A. A. Klusener, A. R. Kucernak, K. E. Jelfs, N. B. McKeown, Q. Song, *Nat. Commun.* **2022**, 13, 3184.
